# T Cells on Engineered Substrates: The Impact of TCR Clustering Is Enhanced by LFA-1 Engagement

**DOI:** 10.3389/fimmu.2018.02085

**Published:** 2018-09-18

**Authors:** Emmanuelle Benard, Jacques A. Nunès, Laurent Limozin, Kheya Sengupta

**Affiliations:** ^1^CNRS, CINaM UMR 7325, Aix-Marseille Université, Marseille, France; ^2^CNRS, UMR7258, Centre de Recherche en Cancerologie de Marseille, Immunity and Cancer Team, Institut Paoli-Calmettes, Inserm, U1068, Aix-Marseille Université UM 105, Marseille, France; ^3^LAI, CNRS UMR 7333, INSERM UMR 1067, Aix-Marseille Université, Marseille, France

**Keywords:** surface bio-engineering, protein nano-patterning, TCR micro-clusters, T cell adhesion, cell spreading, ZAP-70 clusters, actin organization, co-stimulation

## Abstract

We created APC-mimetic synthetic substrates to study the impact of ligand clustering on T cell activation and spreading. The substrates exhibit antibodies directed against the TCR-complex in the form of a patterned array of sub micrometric dots surrounded by a fluid supported lipid bilayer (SLB) which may itself be functionalized with another bio-molecule. We show that for T cell adhesion mediated by T cell receptor (TCR) alone, in the patterned, but not in the corresponding homogeneous controls, the TCR, ZAP-70 and actin are present in the form of clusters or patches that co-localize with the ligand-dots. However, global cell scale parameters like cell area and actin distribution are only weakly impacted by ligand clustering. In presence of ICAM-1 - the ligand of the T cell integrin LFA-1 - on the SLB, the TCR is still clustered due to the patterning of its ligands, but now global parameters are also impacted. The actin organization changes to a peripheral ring, resembling the classical actin distribution seen on homogeneous substrates, the patterned membrane topography disappears and the membrane is flat, whereas the cell area increases significantly. These observations taken together point to a possible pivotal role for LFA-1 in amplifying the effect of TCR-clustering. No such effect is evident for co-engagement of CD28, affected *via* its ligand B7.2. Unlike on ICAM-1, on B7.2 cell spreading and actin organization are similar for homogeneous and patterned substrates. However, TCR and ZAP-70 clusters are still formed in the patterned case. These results indicate complementary role for LFA-1 and CD28 in the regulation and putative coupling of TCR micro-clusters to actin. The engineered substrates presented here clearly have the potential to act as platform for fundamental research in immune cell biology, as well as translational analyses in immunotherapy, for example to screen molecules for their role in T cell adhesion/activation.

## 1. Introduction

The interaction of T cells with antigen presenting cells (APCs) plays a central role in adaptive immunity, one of whose salient features is the duality of exquisite sensitivity and strict discrimination in the context of recognition of antigen by T cells through the T cell receptor (TCR). To achieve this, the T cell membrane carries a variety of additional adhesive, co-stimulatory and inhibitory molecules to complement the basic TCR mediated interaction. Following encounter with its antigen, a key step toward correct activation of the T cell is the reorganization of its membrane and its cytoskeleton. Concomitantly, the cell spreads on the APC, forming the so-called immunological synapse (1, 2). The extent of spreading in T cells is a marker of their activation and eventual proliferation (3).

A very fruitful approach to dissect adhesion and membrane/cytoskeleton reorganization has been to replace the APC with a synthetic antigen presenting substrate (APS). The early APS were, typically, supported bilayers carrying ligands for the TCR/CD3 complex either lipids coupled pMHC (1) or biotinyled anti-CD3 (4), and ligands for the integrin LFA-1 often in the form of a GPI-anchored protein or connected to NTA functionalized lipids (1, 4–6). In the last decade, such substrates have been designed to carry, in addition, a host of other ligands against co-receptors such as CD28 to dissect specific aspects of T cell response.

Seminal work from Dustin and Saito labs underlined the importance of TCR-clusters in initiation of T cell activation (4, 7, 8). Segregation of TCR into clusters is also at the heart of the kinetic segregation model for T cell activation (9–11), where the membrane topography plays an important role. The pair TCR/pMHC being much shorter than LFA-1/ICAM-1 (Intercellular adhesion molecule 1) pair, the TCR clusters exclude the longer LFA-1 / ICAM-1 as well as the long phosphatases like CD45. The absence of CD45 permits the phosphorylation of the ITAM-motifs associated with the TCR/CD3 domain, thus initiating T cell activation.

Simultaneously with activation and adhesion, actin polymerization is triggered at the cell edge to promote spreading (12). The actin is organized roughly as a ring (13), whose fine structure was revealed recently (14–16). It was shown that the peripheral nature of the actin ring is more pronounced for stronger pMHC ligands as compared to weaker ones where the distribution is more homogeneous (14). The TCR as well as LFA-1 get connected to the actin cytoskeleton: the molecular species connecting integrin LFA-1 to actin are well known (17–19), those connecting TCR to actin are yet to be fully identified (17). TCR-clusters that already exclude both LFA-1 and long sugars, are carried by the actin retrograde flow toward the center where they eventually form the central supramolecular activation cluster (cSMAC) (4, 20).

To address the question of the impact of ligand/receptor segregation or clustering two approaches have emerged genetic modification to vary relevant molecular lengths (21, 22), or manipulation of the clustering itself sometimes called spatial mutation (23), for example *via* the use of designed supported lipid bilayers (SLBs) that are patterned with micron size corals which do not allow diffusion of molecules across their fence (20, 24), thus revealing the importance of ligands diffusion for the formation of a stable immunological synapse.

In parallel to the use of SLBs as APS, several groups used protein coated glass instead (13, 25, 26). Using this approach, Irvin and Doh explored the consequences of micro-clustering of TCR and/or LFA-1, focusing on the formation of cSMAC (25). They showed that T-cells can be fully activated when focal spots of immobilized TCR ligand are at the center of the interacting surface but not if they are patterned differently. Later it was shown that T-cells were able to produce IL-12 when anti-CD3 dots are surrounded by CD28 (co-stimulation molecule that binds to B7.1 or B7.2) whereas when both were co-localized they did not (27). These studies emphasized the importance of the organization of the ligands on APC-side for the formation of the immunological synapse and the activation of the T-cells. The importance of force at the synapse is more and more recognized as central (28). A recent study reported complementary roles of TCR and LFA-1 on cytoskletal growth and contractility using micro-patterning showing that LFA-1 adhesion enhances actomyosin forces, which in turn modulate actin assembly downstream of the TCR (29).

In previous work using sub-micron sized patterns of TCR-ligands, we showed that T cells respond globally to average density of TCR-ligands, rather than details of the pattern (30), a result consistent with those obtained with nano-patterns, where the ligand spacing and density were independently controlled (31, 32). However, we could additionally show that on patterned substrates, at the local dot-scale, TCR and ZAP-70 are gathered into clusters that overlap with dots of TCR-ligands.

In many of the examples above, micro and nano patterning of ligands was used to manipulate T cell behavior in order to reveal the importance of TCR clustering. More recently, it has been shown that the natural ligands of TCR, the pMHC ^1^, may in fact be presented as nano-clusters on target cells (33, 34). To this extent, nano-patterned substrates also mimic one aspect of the *in vivo* situation.

Here, as in our previous work (5, 30), the ligand of choice is anti-CD3 which provides sufficient adhesion to the substrate with TCR/CD3 complex alone, in absence of ICAM-1 something not possible if pMHC was used since the TCR-pMHC bond is not strong enough to sustain adhesion. At the same time, it should be pointed out that this is a legitimate approach since anti-CD3 is known to elicit the same signaling pathways as pMHC ligation (35) and the CD3 domain mediates T-cell mechanotransduction (36). We use a combination of colloidal bead lithography and metal sputtering to fabricate sub-micron sized ligand clusters on glass (37, 38). These clusters are then surrounded by supported lipid bilayers, optionally functionalized with ICAM-1 or B7.2 ^2^ to form substrates that mimic APCs. This approach allowed us to simultaneously observe global adhesion as well as local membrane/actin reorganization using high resolution optical microscopy.

## 2. Materials and methods

### 2.1. Substrates preparation

#### 2.1.1. Protein nano-pattern

The details of the fabrication process for making the patterned substrates was published previously (30, 38, 39). Briefly, hydrophilic glass coverslides (thickness = 170 microns, Assistant, Karl Hecht KG, Germany, 24 x 24 mm) were obtained by cleaning by ultrasonication in aqueous solution of a detergent (Hellmanex, Sigma, France), followed by a thorough rinsing in ultrapure water. Self-assembly of colloidal beads was used to create the primary mask. Monodisperse silica colloidal beads (Corpuscular, USA), 2μm diameter, were washed 10 times with ultra-pure water before utilization. The concentration of the beads suspension needs to be optimized in order to avoid multilayer of beads during the deposition. Moreover in order to have an optimal mask, size standard beads was used. A cleaned glass slide was placed on the platform with an angle of about 15° and a calibrated volume of the colloidal suspension was allowed to spread on the slide. After complete evaporation, a large area covering most of the slide of a very ordered array of beads is generated.

A thin and controlled layer of aluminum was deposited on the glass slide through the beads using a radio frequency (RF) magnetron sputtering technique from an aluminum target with 1% silicon (Kurt J. Lesker Company, purity 99.99%). The geometry of the sputtering system is off-axis and the mean free path is 10 mm in the operating pressure range. The samples were placed at a distance of 105 mm onto a rotating table (3–5 rpm). After aluminum deposition, the colloidal beads were rinsed away by ultra-sonication in ultra-pure water to reveal the secondary mask which is the aluminum layer displaying an ordered array of empty nano-holes. The slides were then placed in a chamber containing 3-aminopropyltriethoxysilane (APTES) (Sigma-Aldrich, France) in vapor phase at about 60°C for 1 hour. Next, Bovine Serum Albumin conjugated with biotin (BSA-Biotin, Sigma, France) was incubed at the concentration of 25 μg/ml for 30 min. Finally, the layer of aluminum was removed by incubation in PBS + Sodium hydroxide (NaOH), pH ≥ 11 for at least 4 hours until complete dissolution at room temperature. At this stage the coverslide was covered with uniform nano-dots of functional BSA-Biotin surrounded by bare glass (39).

#### 2.1.2. Preparation of the SLB

The bare glass separating the BSA-biotin dots was filled with a supported lipid bilayer using Langmuir-Blodgett technique [see, for example (5) or (40) for details]. Lipids (Avanti Polar Lipids, USA) were received either dried or already solubilized in chloroform. Dry lipids were dissolved in clean chloroform (99.9%, Sigma, France) before use. Supported lipid bilayers are composed of either pure DOPC (1,2-dioleoyl-sn-glycero-3-phosphocholine), or DOPC+5% NTA-lipids (1,2-dioleoyl-sn-glycero-3-[(N-(5-amino-1-carboxypentyl)iminodiacetic acid)succinyl] (nickel salt)). In order to verify the presence and check the quality of the SLB, 0.01 % of Dansyl PE (1,2-dioleoyl-sn-glycero-3-phosphoethanolamine-N-(5-dimethylamino-1-naphthalenesulfonyl) (ammonium salt)) was systematically added to the mixture.

#### 2.1.3. Functionalization

Substrates were further functionalized by incubation with 2 μg/ml Neutravidin Fluorescein isothiocyanate conjugated (Sigma Aldrich, France) or Neutravidin Dylight650 conjugated, (Life technology, France), (both henceforth called NaV) for 30 min, followed by incubation in anti-CD3 at 2μg/ml (multiniotinylated UCHT1, eBioscience, France) alone or with ICAM-1 His-Tag (ICAM1 Recombinant Human Protein, hIgG1-Fc.His Tag, ThermoFisher, France) or with B7-2 His-Tag (B7-2/CD86 Recombinant Human Protein, R&D systems, France) at 5μg/ml for 30 min.

#### 2.1.4. Homogeneous controls

For homogeneous substrates, the supported lipid bilayer was deposited directly on cleaned glass slides. Two types were used. For negative controls, the SLB composition is the same as described previously and for homogeneous controls, the SLBs were additionally doped with 0.01% cap-biotin (1,2-dipalmitoyl-sn-glycero-3-phosphoethanolamine-N-(cap biotinyl) (sodium salt)) in order to have the same protein composition as on patterned substrates.

### 2.2. Cell culture, fixation and labeling

Jurkat T cells (clone E6-1, ATCC) were cultured in complete RPMI 1640 medium (Life Technologies, France) containing red phenol and L-glutamine supplemented with 1 % glutaMAX (Life Technologies, France) and 10% Fetal Bovine Serum (Life Technologies, France). Cells were in exponential growth phase at the time of experiment. The functionalized glass coverslides formed the bottom of a custom made chamber which was filled with PBS+0,1%BSA buffer. 200 μl of the medium containing cells was added. The cells were allowed to sediment on to the substrate and were incubated for 30 min at 37°C and 5% CO_2_. Cells were then fixed by incubation in 2% pre-warmed paraformaldehyde for 30 min at 37 °C, followed by extensive rinsing with PBS. The cells were blocked with 1% BSA overnight and immunostained by incubation with 5 μg/ml of FITC fluorescent Anti-Vβ8 TCR (BD Biosciences, USA) which is directed against the beta chain of the T-cell receptor, or with 20 μg/ml of Alexa Fluor 488 -phalloidin (dissolved in methanol, ThermoFischer, France) which labeled filamentous actin or with Alexa Fluor 647 Mouse Anti-ZAP70 (PY319)/Syk (PY352) (BD biosciences, France) which labeled the kinase ZAP-70 during 30 min. Samples were rinsed extensively before imaging.

### 2.3. Microscopy

Total internal reflection microscopy (TIRFM) and reflection interference contrast microscopy (RICM) were performed using an inverted microscope (AxioObserver, Zeiss, Germany), equipped with an EM-CCD camera (iXon, Andor, UK). Acquisition was performed using Andor iQ, or Zeiss ZEN software. TIRF and RICM images were taken with a 100X 1.45 NA oil or a custom 100X 1.46 NA oil antiflex objective (Zeiss). For TIRF exposure time was 100 ms, and fluorescence filter sets adapted to the fluorophores were used. For RICM exposure time was 200 ms.

#### 2.3.1. Image analysis

Image analysis was performed using macros written in-house in ImageJ/FIjI (41) and IgorPro (Wavemetrics, USA).

*Supported lipid bilayer*: The presence and the fluidity of the SLB were systematically verified in epi-fluorescence. Quantitative measurements of the fluidity were performed on randomly selected substrates using continuous photobleaching (CPB) (40, 42). Briefly, the fluorescent SLB is continuously illuminated and observed in epi-mode through a partially closed diaphragm, such that the exposed fluorophores are irreversibly bleached. If the SLB is fluid, unbleached fluorophores enter and exit the observation area, resulting in a luminous ring along the edge of the diaphragm. Quantification of the radial profile of this ring yields the lipid diffusion constant.

Note that the SLB fluidity acts as quality check here in our experience fluid bilayers are less prone to defect-formation and phase separation. The proteins however may diffuse with a different diffusion constant that the lipids, the former being usually much slower. In fact, anti-CD3 does not diffuse at all on the SLBs prepared using Langmuir-Blodgett (5). We verified, by measurement of a partial recovery after bleaching, that his-tagged proteins (like ICAM-1 and B7.2 used here) do diffuse, but with a diffusion constant of ≪0.001 μm^2^ - too slow to be quantified by CPB.

*Nano-dots*:The nano-dot were characterized using an automated algorithm in terms of diameter size and fluorescence intensity. The spacing between each dots is set by the diameter of the beads used. For each field of fluorescent nano-dot array, each dot was segmented and a median dot was constructed. The size of the dots was characterized in terms of full-width at half maximum (FWHM) of the radial profile of fluorescence of the median dot. The intensity inside (I_*max*_) and outside (I_*min*_) of the nano-dots corresponding respectively to the value of the intensity of the peak and the value of the baseline intensity on the radial profile. The fluorescence intensity is an important parameter as it is proportional to the protein density. In order to convert fluorescence intensity value of NaV to a estimate density, a calibration was done. For this a special substrate with very low surface density of fluorescent molecules was prepared. The number of fluorescent molecules/μm^2^ was calculated, by assuming that fluorescent dots correspond to one molecule. Then for a corresponding intensity value, the density is known. Therefore, by comparison of the emitted light intensity of experimental substrates, the density of NaV can be determined [also see (5)].

*Cell adhesion area*: Cell adhesion was characterized based on the RICM images in terms of adhesion area. Cell contour was determined from RICM images using a spatial variance filter with a radius of 4 pixels followed by a thresholding. Then the function “Particle analysis" of ImageJ was applied to identify the edge of the cell providing an accurate measurement of the contact area (5, 43). For each condition, a large number of experimental data was available but these had to be additionally vetted to exclude outliers which exhibit atypical behavior probably due to undetected differences in the substrates whose preparation is complex. To systematize this, in addition to discarding substrates for which quantification of the protein density was not available, we also compared data from each sample to an aggregate data-set for the same condition and rejected the data-sets whose p-values showed them to be significantly different (*p* < 0.0001).

*Uniform and textured adhesion*: Casual inspection of the cells RICM images on patterns reveals that two major cell adhesion morphologies are possible (Figure S1). In RICM images, dark areas correspond to tight adhesion and gray areas to the background. Bright pixels however may either arise from the proximal membrane of the cell close to the substrate (typically up to about 800 nm) but not tightly adhered, or sometimes from internal organelles of the cell. The presence of multiple dark patches whose size and spacing matches that of the underlying ligand-dots, points to a cell membrane being textured due to the patterning of the substrate. Here we adapted the convention that presence of at least 7 dark patches (intensity ≪ background), whose size and spacing are compatible with the pattern signify textured adhesion, all others are considered uniform adhesion.

*TCR-clusters*: The fluorescent images of TCR were prepared in ImageJ by doing a segmentation of the cells using the corresponding RICM image, then a intensity thresholding algorithm was used to segment the clusters [The algorithm as a plugin was kindly provided by Dr. Rajat Varma ((6)). The algorithm uses an initial intensity thresholding using the mean intensity under the cell but outside the clusters. Then different parameters were defined : an upper cutoff for cluster size (here 10 pixels), a step value for convergence (here 0.05) and a step value to determine how much to trim each cluster (here 0.8). The algorithm outputs the size of the clusters which was directly used to construct the size histograms [see also (30)). We verified that the output of this algorithm is robust to small (10–25%) changes in the input parameters and the choice of intensity thresholding.

*F-Actin clearance*: In order to determine the organization of the actin (homogeneous or peripheral), fluorescence intensity within a circle of 1.2μm at the center (I_*Center*_) and in the rest (I_*restCell*_) of the cell is measured and then the F-actin clearance is calculated using $\frac{I_{Center}}{I_{RestCell}}$.

*Statistical test and errors*: Error-bars are standard deviations unless otherwise stated. The data were compared pair-wise using 2 tailed Wilcoxon-Mann-Whitney rank test, performed using the in-built function in IgorPro (Wavemetrics, USA). *p*-values were used to determine significance levels. ^***^signifies *P* ≤ 0.0000001, ^**^signifies *P* ≤ 0.0001, ^*^ signifies *P* ≤ 0.01 and NS (or absence of any ^*^) signifies >0.01. In addition, within populations deemed to be different, we quantify the size of the difference (effect-size) through the difference in the medians.

## 3. Results

### 3.1. The substrates

**Figure 1:**
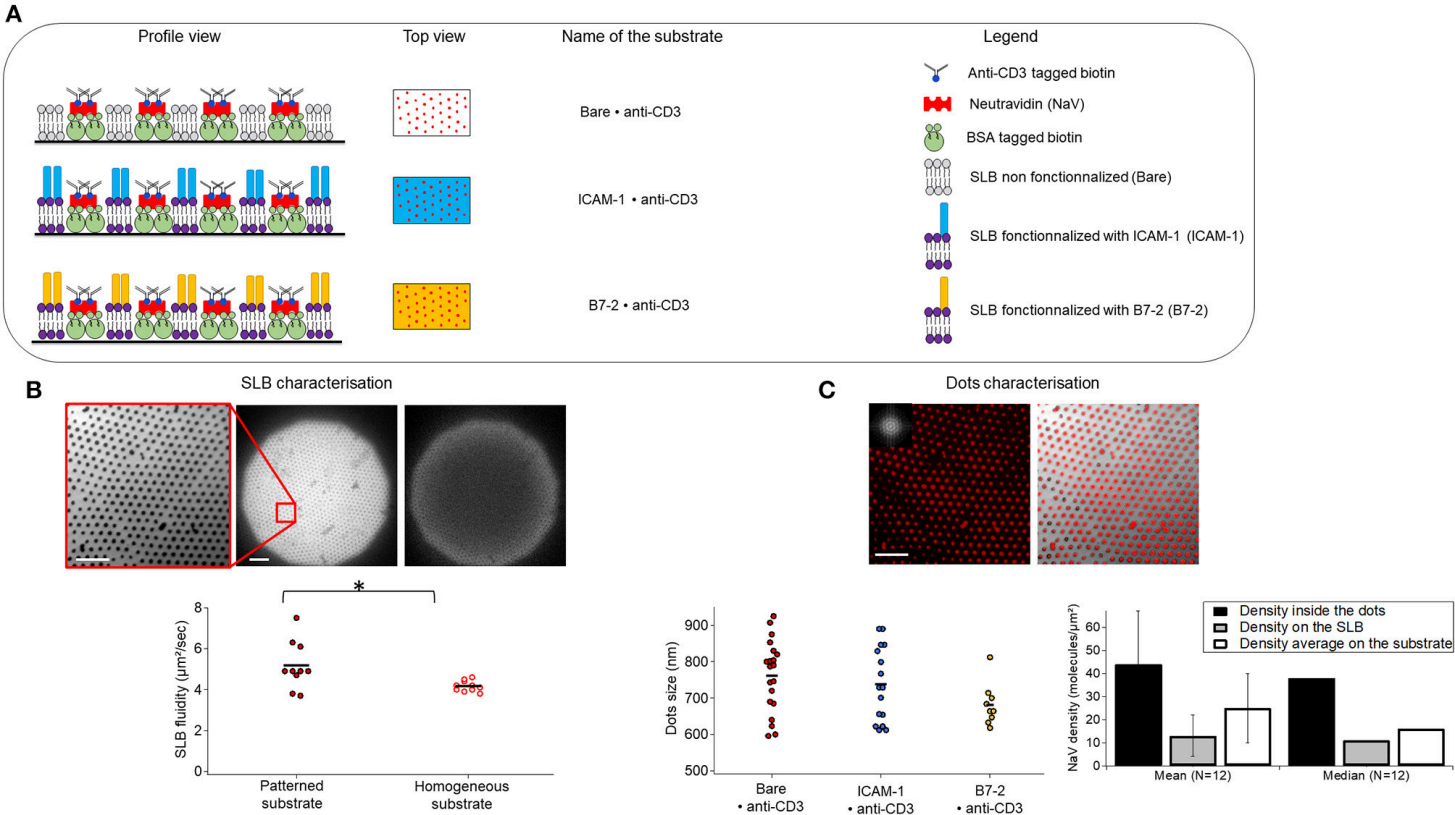
Substrate composition and characteristics. **(A)** Schematics of the patterned substrates. Substrates are composed of biotinylated anti-CD3 dots surrounded by a supported lipid bilayer (SLB) which is bare (top), or functionalized either with ICAM-1 (middle) or B7-2 (bottom). Profile and top view are represented. Note that the drawing is not to scale. **(B)** SLB characterization. Left to right: epi-fluorescence image of DOPC bilayer doped with 1% fluorescent lipids surrounding the dots, zoom and full field, and the latter after 50 s of continuous photo-bleaching (here the diffusion coefficient was measured to 5 μm^2^/s), scatter-dot plot of measured diffusion constant for the SLB on homogeneous and patterned substrates. The mean diffusion constant was 4.2 ±0.3 μm^2^/s and 5.2 ± 1.2μm^2^/s on homogeneous (*N* = 10) and patterned substrates (*N* = 10) respectively. N is number of samples, ^*^signifies 0.01 ≤ P ≤ 0.05. **(C)** Characterization of dots. Left to right: epi-fluorescent image of nano-dot arrays of NaV labeled with Dyelight650 (inset is FFT showing the excellent ordering of the lattice), composite image of nano-dot array and the SLB showing the good complementary of the SLB and the protein dots, quantification of NaV dot-size (each point is one sample, 20, 16, and 9 samples for Bare, ICAM-1 and B7.2 respectively, each sample represents about a thousand dots) and density (averaged over 12 samples, each representing thousands of dots, error bars are standard deviation). Results are pooled from up to 70 samples, cumulating thousands of dots. Scale bar = 10μm.

The APC mimetic substrates were prepared using nano-sphere lithography and aluminum sputtering as discussed above. The basic patterned substrate consists of anti-CD3 dots, arranged in a hexagonal pattern and surrounded by a SLB, which can be optionality functionalized with either ICAM-1 or B7.2 (Figure 1A). For ease of reference, we adopt the following nomenclature: anti-CD3 clusters embedded in bare/ICAM-1/B7.2-functionalized SLB are called Bare/ICAM-1/B7.2•anti-CD3. For each type of ligand, patterned substrates were compared to equivalent homogeneous substrates, which are SLBs on which the anti-CD3, conjugated *via* biotin/neutravidin is immobile (5) but the ICAM-1/B7.2, conjugated *via* his-tag/NTA is mobile. The corresponding homogeneous substrates are called Bare/ICAM-1/B7.2 + anti-CD3. The distance between the dots (pitch) is set by the choice of the beads for lithography, here 2μm. The fluidity of the SLB was quantified using continuous photo-bleaching and was found to vary from about 4 to 8 μm^2^/s (Figure 1B). The dot size is fixed by shadow effects during sputtering (38). The dot size and the density of ligands inside or outside the dots was quantified by analysis of epi-fluorescence images of the neutravidin using self-written routines that allowed us to easily analyze thousands of dots (Figure 1C). At about 700–800 nm, the size of the ligand-dots happen to roughly match the typical size of signaling micro-clusters reported in literature (6). The average density of ligands inside a dot was estimated to be about 40 molecules/μm^2^ and outside at about 10 molecules/μm^2^, yielding a good contrast. The average ligand density under a cell was about 20 molecules/μm^2^.

The variation of the density from substrate to substrate as well as within each substrate could potentially impact the results. Since data from a large number of substrates are pooled, it was necessary to verify that the density of the ligands does not impact cell spreading area in the studied range. Indeed as can be seen in Figures S2A–C, this is the case for both patterned and homogeneous substrates, with or without additional functionalization with ICAM-1/B7-2.

Jurkat T-cells were allowed to interact with these APC mimetic substrates for 30 minutes and were fixed. They were then imaged in RICM, and/or TIRF-M. The cell response was quantified through the analyses of the cell spreading area (also called adhesion area), the T-cell membrane molecular distribution (TCR and ZAP-70), and the actin organization. Five parameters are discussed: the cell spreading area, which is a measure of cell activation in T cells (3), TCR/ZAP-70 clustering and membrane topography which together is thought to be essential for activation (9) and actin architecture which plays an important role in molecular transport (17) as well as mechanosensing (36).

### 3.2. Cell spreading and actin organization on patterned and homogeneous anti-CD3

**Figure 2:**
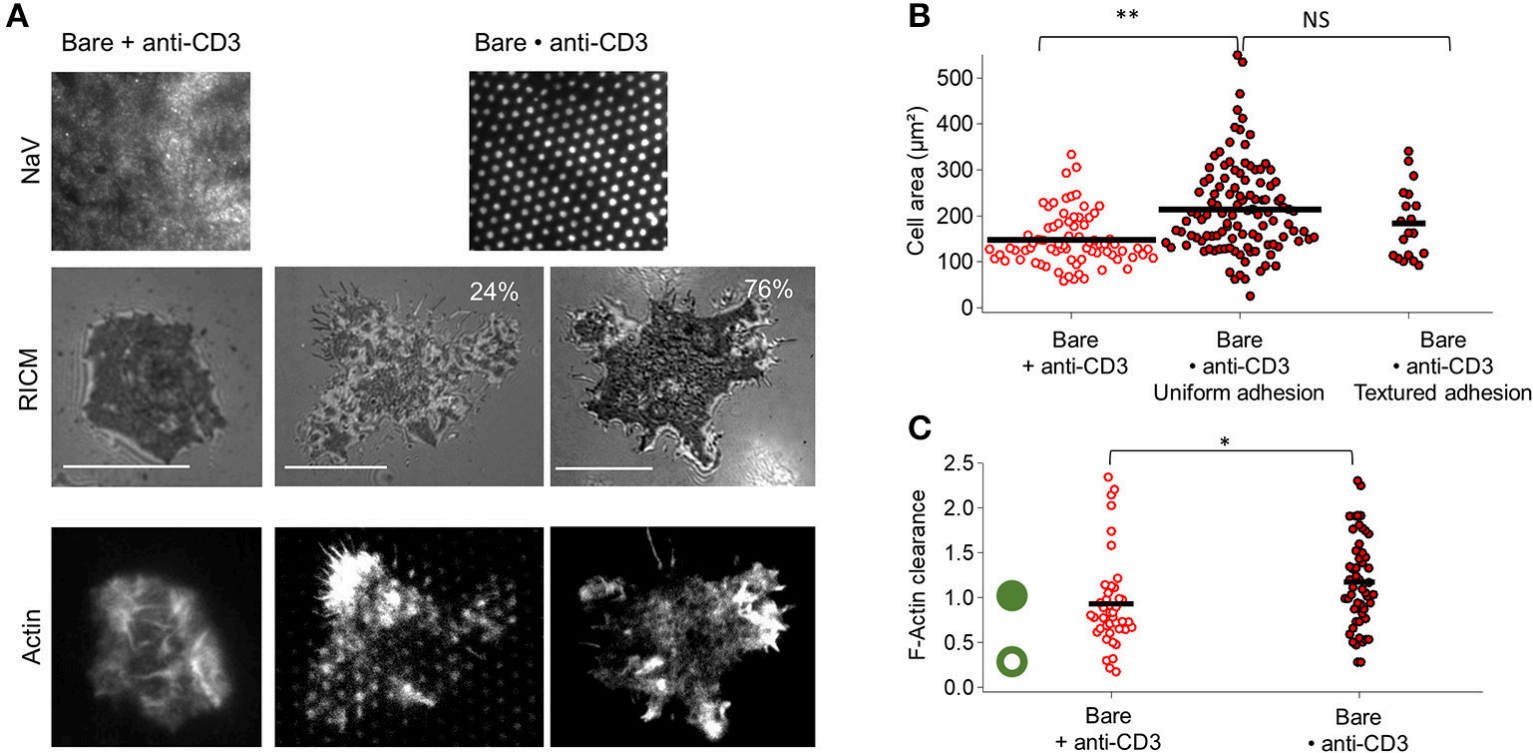
Impact of anti-CD3 clustering on cell adhesion and actin organization. Cells were allowed to interact with homogeneous and patterned substrates, and were fixed and labeled with fluorescent phalloidin. **(A)** T-cell adhesion and actin organization. Top to bottom: epi-fluorescent images of the underlying protein (NaV), RICM images, and TIRFM images of the actin-labeled cells. For patterned substrates, two types of adhesion are shown: ‘patterned’ adhesion seen in about quarter of the cases and ‘uniform’ adhesion seen in the rest of the cases. Scale bar = 10μm. **(B)** Scatter-dot plot of cell spreading area measured from RICM images (*N* = 4, *n* = 75 for homogeneous and *N* = 8, *n* = 109 for patterned). **(C)** Scatter-dot plot of the F-actin clearance calculated from TIRFM images (*N* = 2, *n* = 41 for homogeneous and *N* = 5, *n* = 52 for patterned). Green ring/disc schematizes actin architecture for corresponding clearance parameter. Bar is at mean value. *N* is number of samples and *n* is total number of cells.

For anti-CD3 dots embedded in bare SLB (Bare•anti-CD3), casual inspection of RICM images reveals that on Bare•anti-CD3 two major cell adhesion morphologies are possible—either uniform or textured (Figure 2A). In the first case, true for about 75–80% of the cells, the cell membrane adheres uniformly to the underlying substrate; and in the second case, true for about 20–25% of the cells, the membrane is textured (Figure 2, Figure S1). The cell spreading area for the two types of adhesion (184 ± 78 μm^2^ for textured adhesion and 214 ± 97 μm^2^ for uniform adhesion) is not statistically different (Figure 2B). Averaging over all cells on Bare•anti-CD3, spreading area is 207 ± 95 μm^2^ (average ± s.d, from 8 experiments, totaling 109 cells, SEM=9 and median = 170 μm^2^), to be compared to spreading area for homogeneously partitioned anti-CD3 grafted on an SLB (148 ± 57 μm^2^, SEM = 7, meadian = 130 μm^2^). The two distributions are statistically different and the effect-size is 40 μm^2^ (see Tables S1, S2 and Figure S3).

The spreading of a T cell is driven by actin polymerization (5, 44) and therefore the adhesion and extent of spreading can be expected to be intimately linked with actin organization. In the classical case of T cells spreading on SLBs functionalized homogeneously with anti-CD3 and ICAM-1, at full spreading, the actin forms a ring along the periphery of the cell (5, 44, 45). In the present case of Bare•anti-CD3 however, the actin is either homogeneous (75–80% of the cells) or appears as dots that clearly coincide with the pattern (20–25%). Quantification of the extent of actin clearance from the center shows that cells on Bare•anti-CD3 and Bare + anti-CD3 are similar in terms of actin clearance (Figure 2C, Figure S4).

### 3.3. Cell spreading and actin organization when LFA-1 integrins or CD28 co-receptors are engaged

**Figure 3:**
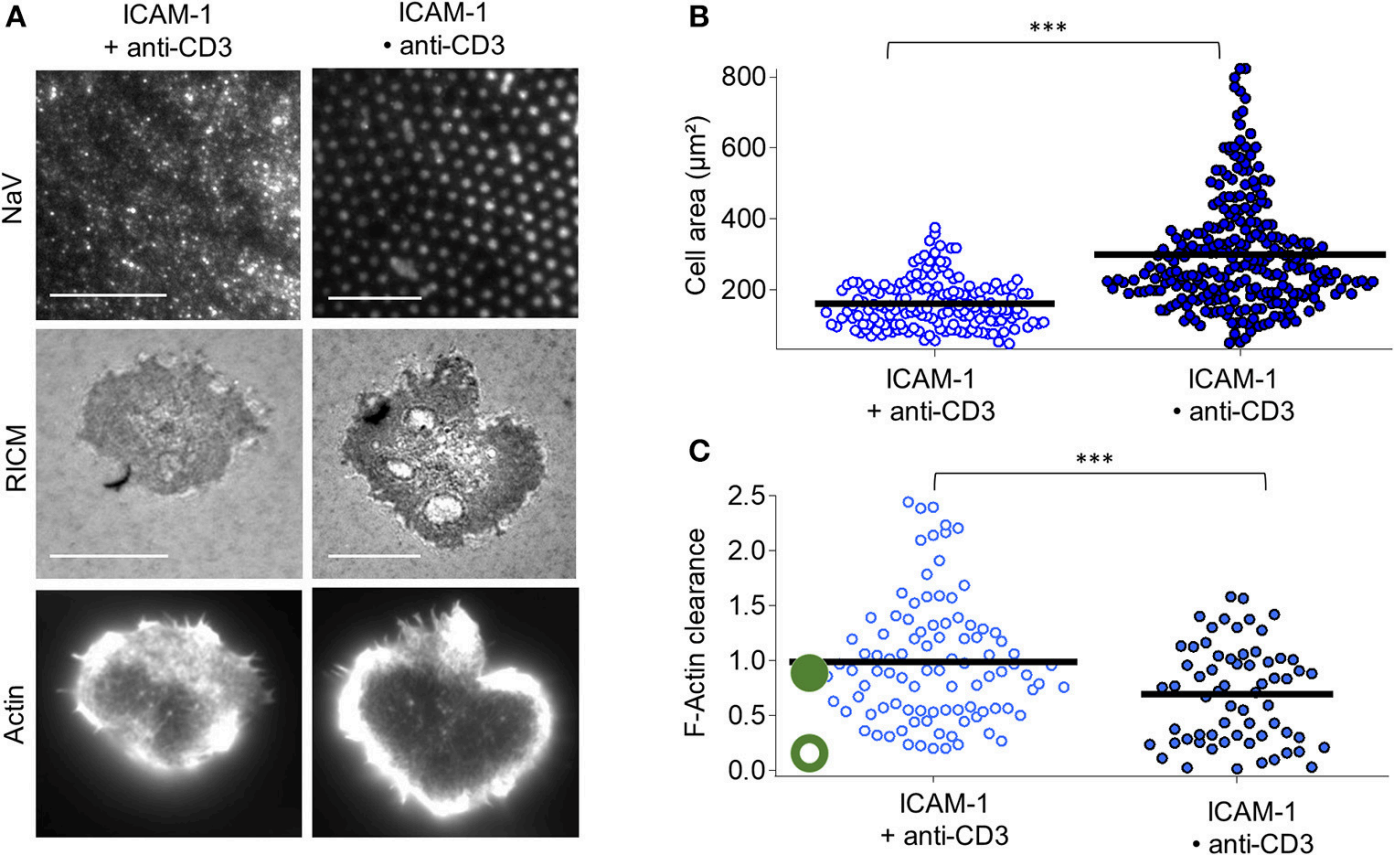
Impact of anti-CD3 clustering on cell adhesion and actin organization in presence of ICAM-1. Cells were allowed to interact with homogeneous and patterned substrates, and were fixed and labeled with fluorescent phalloidin. **(A)** T-cell adhesion and actin organization. Top to bottom: epi-fluorescent images of the underlying protein (NaV), RICM, and TIRFM images of the actin-labeled cells. Scale bar = 10μm. **(B)** Scatter-dot plot of cell spreading area measured from RICM images (*N* = 7, *n* = 164 for homogeneous and *N* = 12, *n* = 271 for patterned). **(C)** Scatter-dot plot of the F-actin clearance calculated from TIRFM images (*N* = 4, *n* = 97 for homogeneous and *N* = 4, *n* = 64 for patterned). Bar is mean value.

For the case of anti-CD3 clusters embedded in ICAM-1 functionalized SLB (ICAM•anti-CD3), RICM images reveal that the membrane adheres uniformly to the substrate for all cells and the substrate patterning has no discernible impact on the membrane roughness (Figure 3A). Indeed, the RICM images of cells on ICAM•anti-CD3 are not qualitatively different from those on ICAM + anti-CD3. The cell area however, is significantly larger (Figure 3B, Tables S1, S2). Averaging over all cells on ICAM•anti-CD3, spreading area is 298 ± 153 μm^2^ (average ± s.d, from 12 experiments, totaling 171 cells, SEM = 9 and median = 259 μm^2^), to be compared to spreading area for homogeneously partitioned anti-CD3 grafted on an ICAM-1 bearing SLB (from 7 experiments and 164 cells, 160 ± 68 μm^2^, SEM = 5, median = 147 μm^2^). The effect-size quantified by difference in median is 111 μm^2^, much higher than in absence of ICAM-1. A comparison between the patterned substrates with and without ICAM-1 (Bare•anti-CD3 and ICAM•anti-CD3), also reveals a significant difference, with the effect-size being 70 μm^2^. We conclude that the presence of ICAM-1 enhances the impact of the clustering of the TCR.

Visual inspection as well as quantification of actin clearance show that the actin organization for cells on ICAM•anti-CD3 is clearly peripheral whereas on ICAM + anti-CD3, a range of behavior from fairly homogeneous to peripheral is seen (Figure 3C). As anticipated, ICAM-1 alone, in the form of dots or not, does not induce cell spreading (Figure S5).

**Figure 4:**
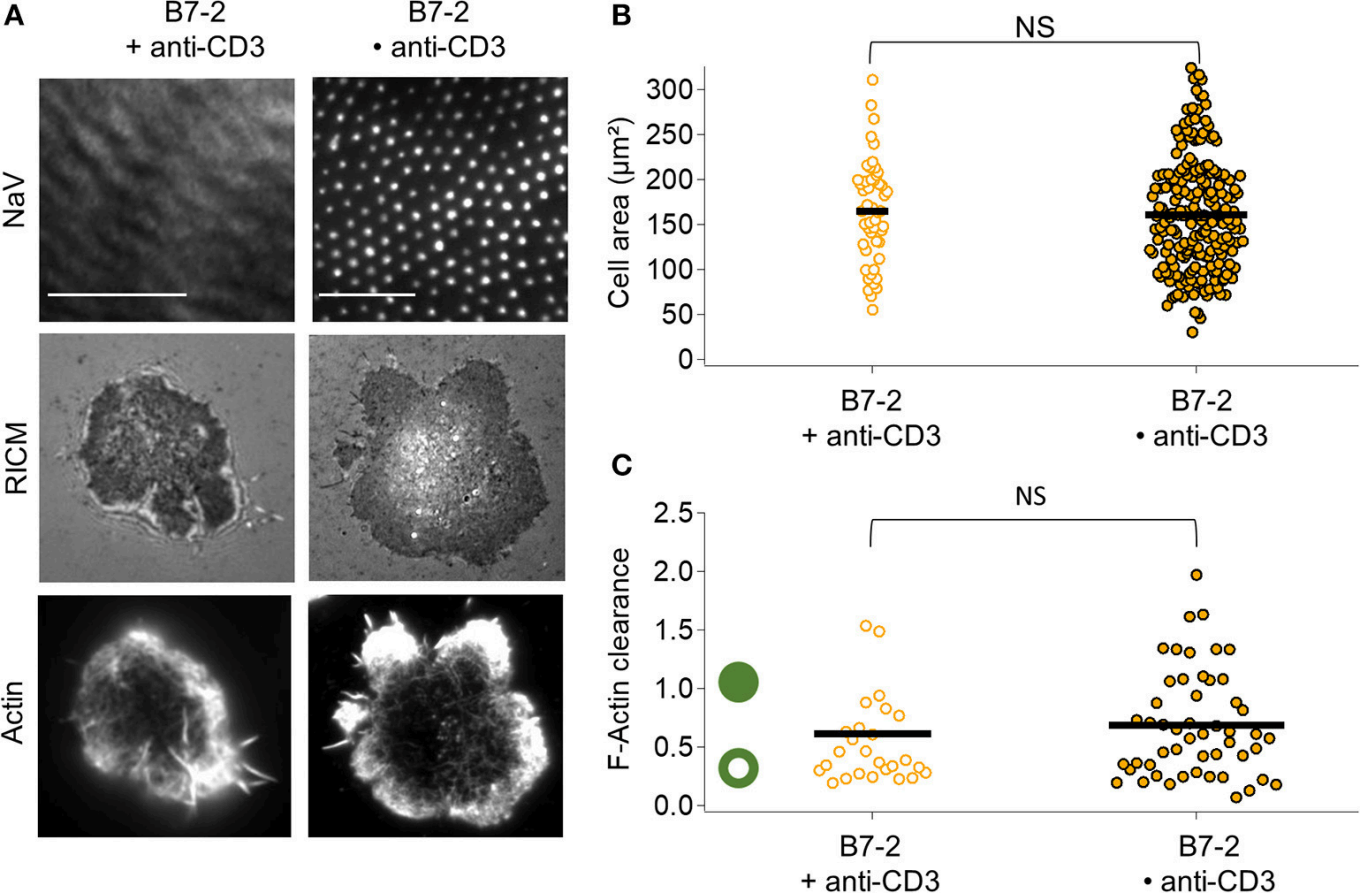
Impact of anti-CD3 clustering on cell adhesion and actin organization in presence of B7-2. Cells were allowed to interact with homogeneous and patterned substrates, and were fixed and labeled with fluorescent phalloidin. **(A)** T-cell adhesion and actin organization. Top to bottom: epi-fluorescent images of the underlying protein (NaV), RICM, and TIRFM images of the labeled cells. Scale bar = 10μm. **(B)** Scatter-dot plot of cell spreading area measured from RICM images (*N* = 2, *n* = 50 for homogeneous and *N* = 11, *n* = 203 for patterned). **(C)** Scatter-dot plot of the F-actin clearance calculated from TIRFM images (*N* = 2, *n* = 26 for homogeneous and *N* = 4, *n* = 51 for patterned). Bar is median value. NS signifies *P* ≥ 0.05.

In presence of B7.2, comparing B7.2•anti-CD3 and B7 + anti-CD3, no statistical difference is detected either in terms of adhesion area or in terms of actin clearance (Figure 4). In both cases, the adhesion is homogeneous, and actin is peripheral.

### 3.4. TCR and ZAP-70 organization

**Figure 5:**
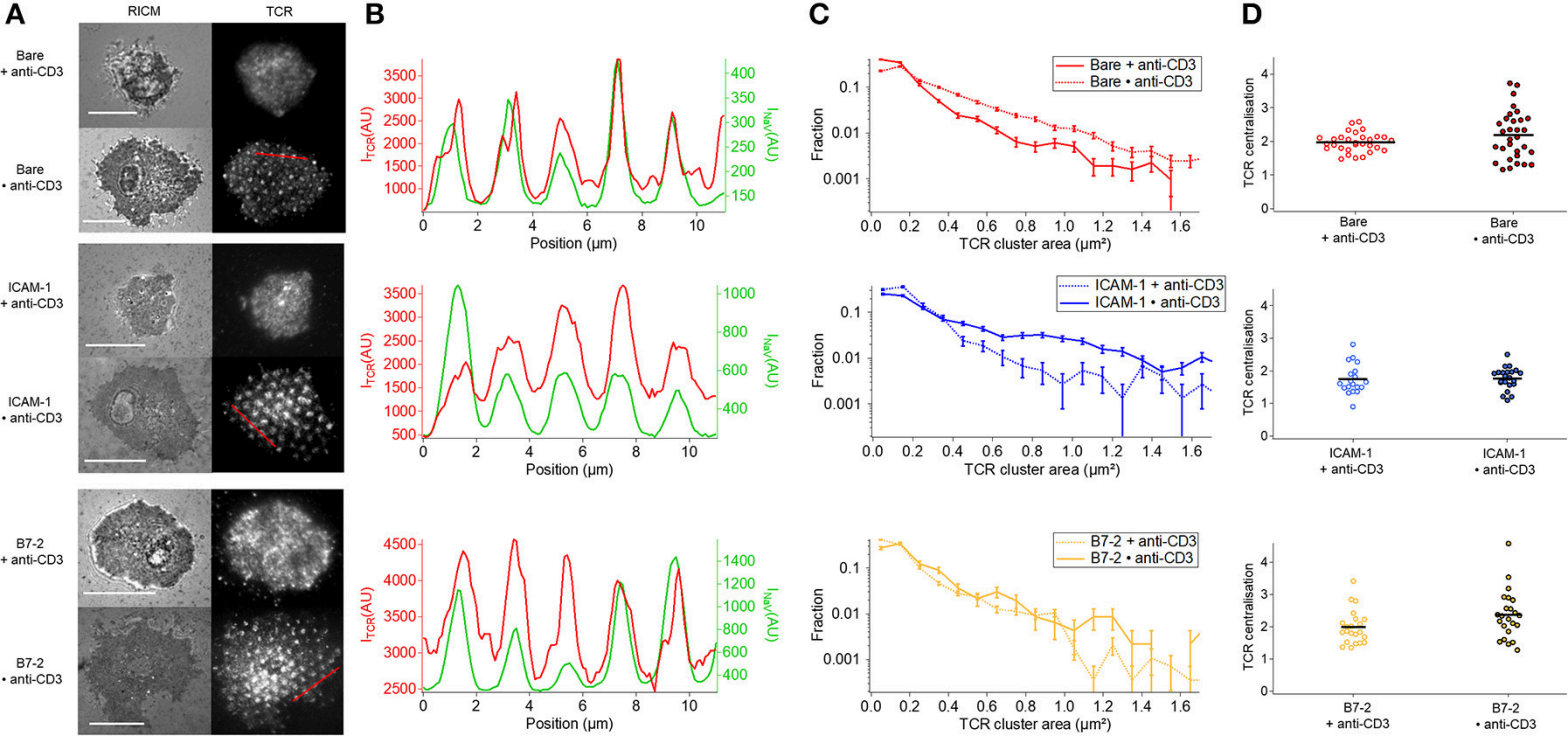
Impact of anti-CD3 clustering on the distribution of T cell receptor (TCR) in absence or presence of ICAM-1 or B7-2. Cells were allowed to interact during 30 min with the substrate, and were fixed and labeled with a fluorescent antibody against TCR. **(A)** Selected examples of RICM and TIRFM images of the TCR-labeled cells interacting with homogeneous (top) or patterned (bottom) substrates. Scale bar = 10μm. **(B)** Line-profile showing the superposition of TCR (red) and underlying dots (NaV, green), see A for the position of the line. **(C)** Histograms of the apparent area of clusters normalized by the total number of clusters. For each case, the data presented corresponds to 1 or 2 experiments, number of cells vary from 7 to 17, and the number of analyzed clusters from about 700 to 4,000. In each case, larger clusters are more numerous on the patterned substrates, compared to the homogeneous counterpart. **(D)** Scatter-dot plot of the TCR centralization calculated from TIRFM images. No centralization is detected. No ^*^s are indicated since *P* ≥ 0.05 for all cases. Error bars are normalized standard deviation.

The co-localization of TCR with anti-CD3 dots occurs in all cases where anti-CD3 is in form of dots (Figure 5A). Whereas on homogeneous anti-CD3, with or without additional presence of ICAM-1 or B7.2, the anti-CD3 is distributed all over the surface and the TCR get bound and immobilized uniformly, on dot anti-CD3 (Bare•anti-CD3 with or without ICAM-1/B7.2) a pool of diffusive and non-bound TCR molecules may be able to diffuse over the SLB and co-localize with the anti-CD3 dots (Figure 5A). On ICAM-1•anti-CD3 and B7.2•anti-CD3, the ICAM-1 or B7.2 can diffuse, and do not hinder the diffusion of TCR. As is seen in Figure 5B, the coincidence of the TCR clusters with the dots is near perfect even though there is no correlation between the ligand density in the dots and the TCR density (reflected by the fact that faint dots may harbor bright clusters and vice versa). Figure 5C shows [after (6)] the distribution of cluster size *via* the fraction of clusters present for each size (also see Table S3A). In each case, an enrichment of large clusters (size greater than, or of the order of ligand-dot size) is evident in the patterned substrate with respect to the homogeneous one. Finally, at the cell scale, the TCR clusters are distributed uniformly, without the formation of an evident cSMAC (Figure 5D). This is quantified *via* the TCR parameter, which was found to be >3 for T cells on SLBs with mobile ligands (5, 30).

**Figure 6:**
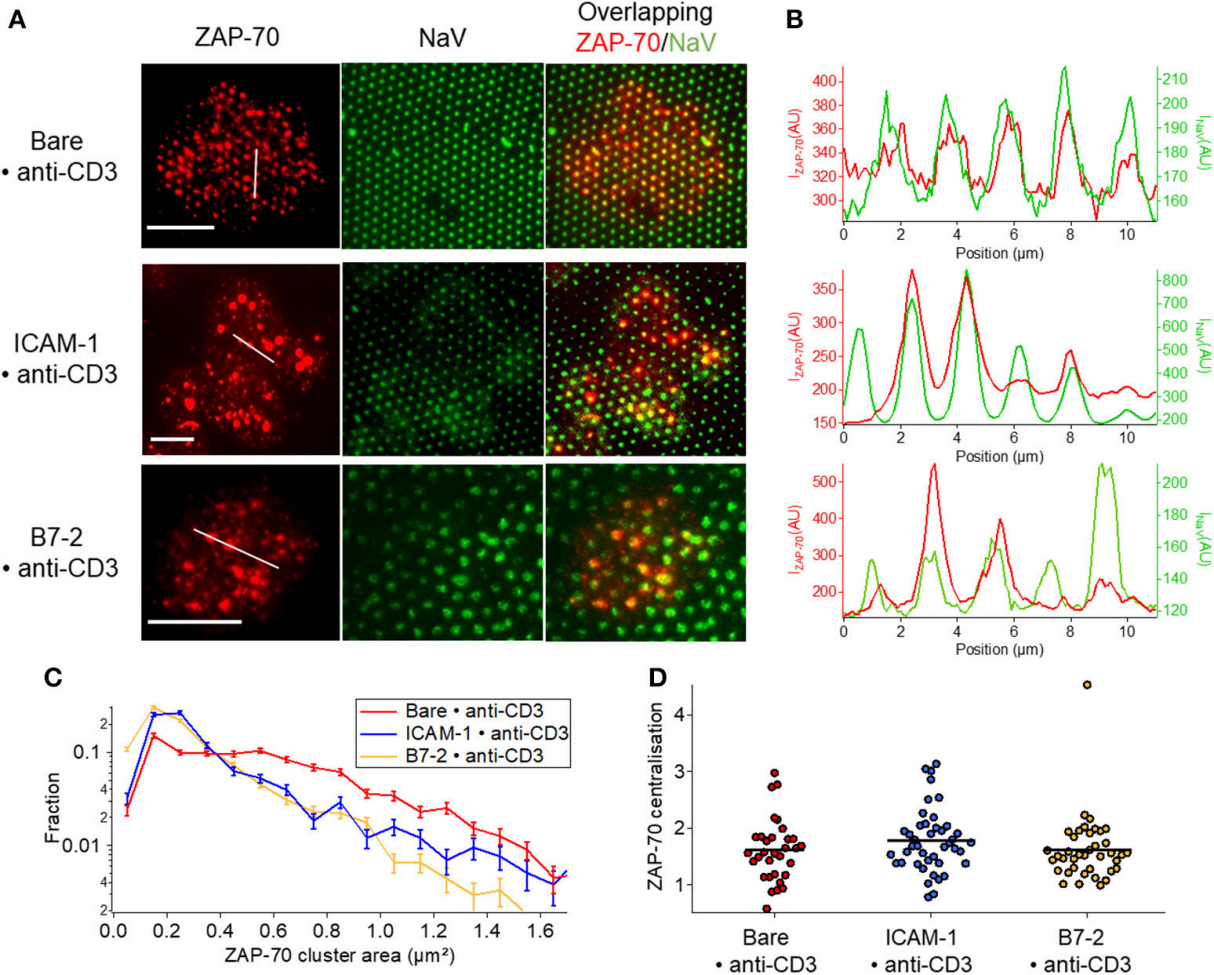
Impact of anti-CD3 clustering on the distribution of ZAP-70 in presence of ICAM-1 or B7-2. Cells were allowed to interact during 30 min with patterned substrates, then fixed and labeled with a fluorescent antibody against the ZAP-70. **(A)** Selected examples of TIRFM images of the marked cells interacting with patterned substrate. Column 1: TIRFM images of the ZAP-70 clusters. Column 2: The underlying fluorescent (NaV) dots. Column 3 : a superposition of the ZAP-70 channel and the NaV channel. Scale bar = 10μm. **(B)** Line-profile showing the superposition of the ZAP (red) and the underlying dot (NaV, green). **(C)** Histogram of the apparent area of clusters normalized by the total number of clusters. Larger clusters, that are co-localized with the dots, are relatively more numerous on Bare•anti-CD3; on ICAM-1•anti-CD3 and B7.2•anti-CD3, in addition to such large clusters, numerous small clusters are also present on the SLB. **(D)** Scatter-dot plot of the TCR centralization calculated from TIRFM images. No centralization is detected. No ^*^s are indicated since P ≥ 0.05 for all cases. Error bars are normalized standard deviation.

ZAP-70 clusters mostly follow the pattern on Bare•anti-CD3 whereas on ICAM-1•anti-CD3 the overlap is relatively poor (Figures 6A,B, Figure S6). Moreover, in presence of ICAM-1 or B7.2, the proportion of ZAP-70 molecules homogeneously distributed over the cell membrane in the form of small clusters (much smaller than ligand-dot size) is higher (Figure 6C, Table S3B). Note the much more pronounced co-localization of the TCR clusters (Figure 5) with the underlying pattern (see also Figure S6 for quantification). These observations are consistent with the plot of cluster size distribution (Figure 6C). Like TCR, no centralization of ZAP-70 was detected (Figure 6D).

## 4. Discussion

Here we presented single cell experiments to explore the link between formation of sub-micron scale TCR clusters and the response at both the local cluster-scale and the global cell-scale. In agreement with our previous work (30, 37), where a passive polymer (PLL-PEG, poly L lysine-polyethylene glycol), rather than a SLB covered the area between the anti-CD3 dots, we showed that when ligands of TCR-complex are immobilized to form of dots or clusters, the TCR themselves form corresponding clusters. The importance of micro-clusters of TCR (4, 7, 8) is now well established and traditionally, it was considered that a SLB containing mobile ligands is necessary to generate micro-clusters on artificial APCs (4). However, unlike in a continuous SLB, the patterned substrates do not allow the centralization of the TCR, the micro-clusters are arrested on top of the pattern, somewhat reminiscent of their confinement on corralled SLBs (20, 24). The centralization of TCR occurs when TCR molecules are coupled to the retrograde actin flow which draws them backwards toward the centre of the cell (20, 46). Here, unligated TCR molecules diffuse freely till they meet their immobilized ligands and then they themselves get immobilized according to the pattern of the underlying ligands. The lack of centralization here shows that unligated TCR does not couple to any retrograde actin flow that may exist.

The significance of micro-clusters have long been debated in literature, and the consensus is converging toward the view that they act as platforms from which long phosphatases are excluded, thus allowing phospholylation of signaling molecules (47). Recently, elegant use of micro-nano patterning that presented or not a surface topography, and therefore presumably induced or not a corresponding texturing of the membrane, strengthened this view (48). In our experiments however, the connection between cell spreading, as a marker of activation, and membrane topography as detected by RICM, is not clear. Strikingly, here we show that cell spreading and actin organization is only weakly impacted by TCR clustering alone, it is the presence of ICAM-1 (but not B7.2) that renders clustering important (Tables S1, S2, Figures S7A,B).

In all patterning studies, in addition to the activating molecules, the means of passivation out of the active zones becomes important (49), as emphasized by us previously (30). In Dillard et al. (30), the surface density of the passivating polymer (PLL-PEG) was varied and it was shown that this has a major impact on cell spreading but only a minor impact on local TCR or ZAP-70 clustering. In the current SLB system too this effect is evident–if the SLB is additionally doped with PEG-carrying lipids, the cells spread less and they do so more slowly. Interestingly, the local membrane topography and gathering of TCR are not impacted (Figure S8).

Here, by using biotinylated lipids and biotinylated BSA interchangeably, we could compare each pattern type with its equivalent homogeneous SLB substrate. In the range of density probed here, the additional presence of ICAM-1 in the homogeneous case did not affect cell spreading (compare bare+anti-CD3 and ICAM+anti-CD3). ICAM-1 however dramatically increased the adhesion area in the patterned case. Cell spread more on ICAM•anti-CD3 both with respect to bare•anti-CD3 as well as ICAM+anti-CD3. This observation points to a possible crucial role for ICAM-1 in amplifying the effect of TCR-clustering. In fact, it has been reported that TCR micro-clusters form “mini-synapses” where the central core is surrounded by a ring of LFA-1 (50). One possible role of this ring is to compactify the clusters to render them denser, consistent with reports that only TCR-dense clusters are signaling-competent (51). Interestingly pre-labeling of the TCR with an antibody (anti-Vβ8) in solution prior to spreading on bare•anti-CD3 leads to highly augmented spreading, perhaps because the antibody promotes cross-linking and cluster compaction (Figure S9). Experiments where the ICAM-1 is presented as dots and with anti-CD3 on the SLB shows that clustering of ICAM-1 has no impact (Figure S5).

However, CD28 co-stimulation of the T cell has a very different impact, as could be expected (52–54). Both on B7.2 + anti-CD3 and B7.2•anti-CD3, the cells spread less as compared to all other cases reported. This may be related to previous reports which showed that CD28 inhibits cell spreading by acting on LFA-1 related down-stream signaling (55). Here, a similar effect seems to be operational even though LFA-1 ligands are not present. Importantly, there was also no difference between B7.2 + anti-CD3 and B7.2•anti-CD3, showing that co-stimulation of CD28 has no effect on amplification of TCR-clustering induced activation. Very interestingly, even though cell spreading is limited, the actin forms a ring, contrary to expectations based on other studies where actin ring was associated with enhanced spreading (12).

Globally, considering all the substrates together (Figure S7), the enhancement in activation (as quantified by cell spreading and corroborated by actin ring formation) on ICAM1•anti-CD3 as compared to all the other substrates, cannot be explained by topographical differences and CD45 expulsion alone ^3^. Soliciting LFA-1 but not CD28 triggers enhanced spreading even though the actin organization is similar in both cases and retrograde flow is present (data not shown). The friction model of spreading (5), links the actin retrograde flow and its coupling to ligand/receptor kinetics to cell spreading area. Presence of retrograde flow but diminished spreading would indicate a lack of transfer of traction to the substrate in case of CD28, leading us to infer that the link between the TCR-complex (*via* CD3) and actin is enhanced by activation of LFA-1 and weakened by ligation of CD28. Thus while co-stimulation by CD28 and engagement of LFA-1 are used to activate/adhere T cells, the impact on spreading is complementary and the two together may regulate the engagement of TCR with actin, a step crucial for later centralization of the TCR into cSMAC.

## 5. Conclusion

Using our engineered substrates, we have evidenced the role of integrins in enhancing the impact of TCR clustering on cell spreading and actin organization, and shown that the co-receptor CD28 has no such role in amplification of the effect of TCR-clustering. The work presented here underlines the potential of nano-patterned substrates to decipher fundamental questions in T cell biology. Engineering the interface, combined with genetic engineering of the cell, can become a powerful and indispensable tool in immunobiology and be adapted for improved translational devices in immunotherapy.

## Author contributions

EB did experiments and analysis. KS and LL conceived and directed the project and participated in data analysis. EB, JN, LL, and KS interpreted the results. KS and EB wrote the manuscript.

### Conflict of interest statement

The authors declare that the research was conducted in the absence of any commercial or financial relationships that could be construed as a potential conflict of interest.

## Footnotes

^1^So far the reports concern pMHC-II.

^2^CD28 interacts with two major ligands, B7.1 and B7.2 with no significant differences in the functions of CD28/B7.1 versus CD28/B7.2. For this study, we used B7.2 as it is generally the first B7 molecule encountered, due to its constitutive expression on numerous antigen presenting cells.

^3^The height difference between dots and SLB for Bare•anti-CD3 is about 27 nm dots being taller, for ICAM-1•anti-CD3 is about 15 nm, dots being shorter and for B7.2•anti-CD3 again 15 nm but with dots being taller.
