# Supplementary material for: T Cells on Engineered Substrates: The Impact of TCR Clustering Is Enhanced by LFA-1 Engagement
Source: Front Immunol. 2018 Sep 18;9:2085. doi: 10.3389/fimmu.2018.02085 (PMC6154019; doi:10.3389/fimmu.2018.02085)
Supplement: Supplementary file 1 [file Data_Sheet_1.pdf]

Supporting information

T cells on engineered substrates: the impact of TCR clustering is enhanced by LFA-1 engagement

Emmanuelle Benard, Jacques A. Nunes, Laurent Limozin and Kheya Sengupta

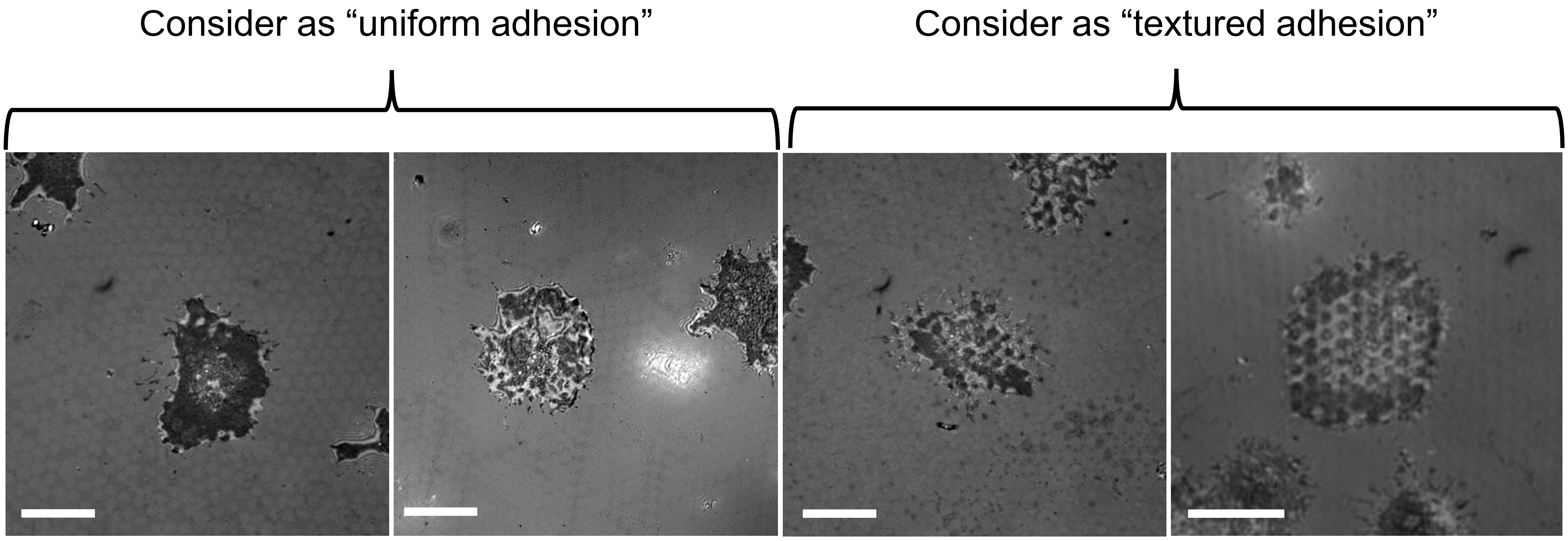

Fig S1 : **Examples of cells exhibiting “uniform adhesion” or “textured adhesion”**. In the RICM images, dark areas correspond to tight adhesion and gray areas to the background. Bright pixels however may either arise from the proximal membrane of the cell close to the substrate (typically up to about 800 nm) but not tightly adhered, or sometimes (as in the example of the central zone of the left-most cell), from internal organelles of the cell. This dual origin of brightness makes it difficult to fully automatize the detection of membrane undulation/texture. However, the presence of multiple dark patches whose size and spacing matches that of the underlying ligand-dots, points to a cell membrane being textured due to the patterning of the substrate. Here we adapted the convention that presence of at least 7 dark patches (intensity << background), whose size and spacing are compatible with the pattern signify “textured adhesion” (example the two right-most cells), all others are considered “uniform adhesion” (example the two left-most cells).

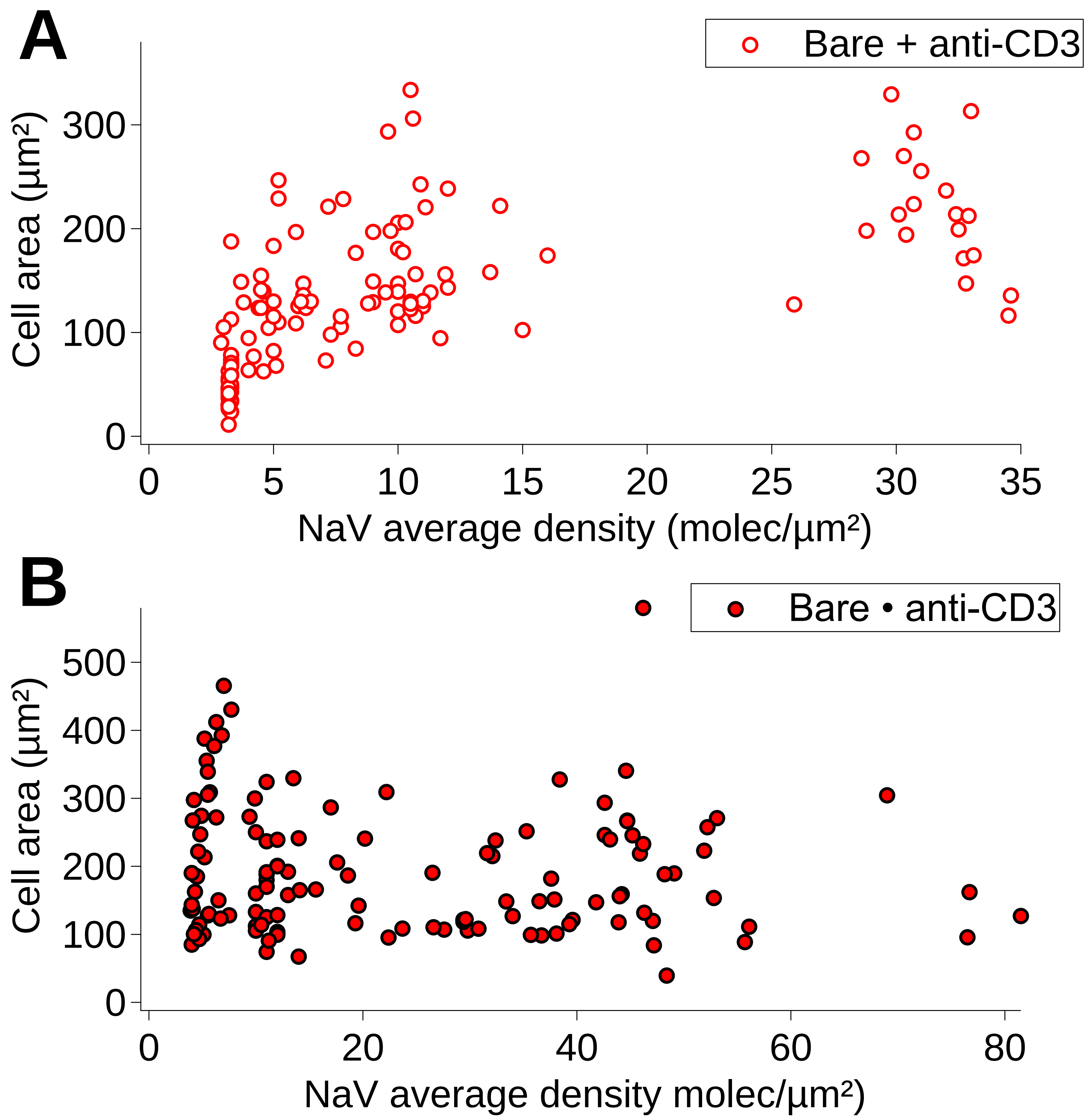

Fig S2A: **Impact of NaV density on cell spreading area.** Cell adhesion area as a function of the average NaV density over the whole sample, on homogeneous (**A**) or patterned (**B**) substrates fonctionnalized with anti-CD3 alone. Each point represent one cells.

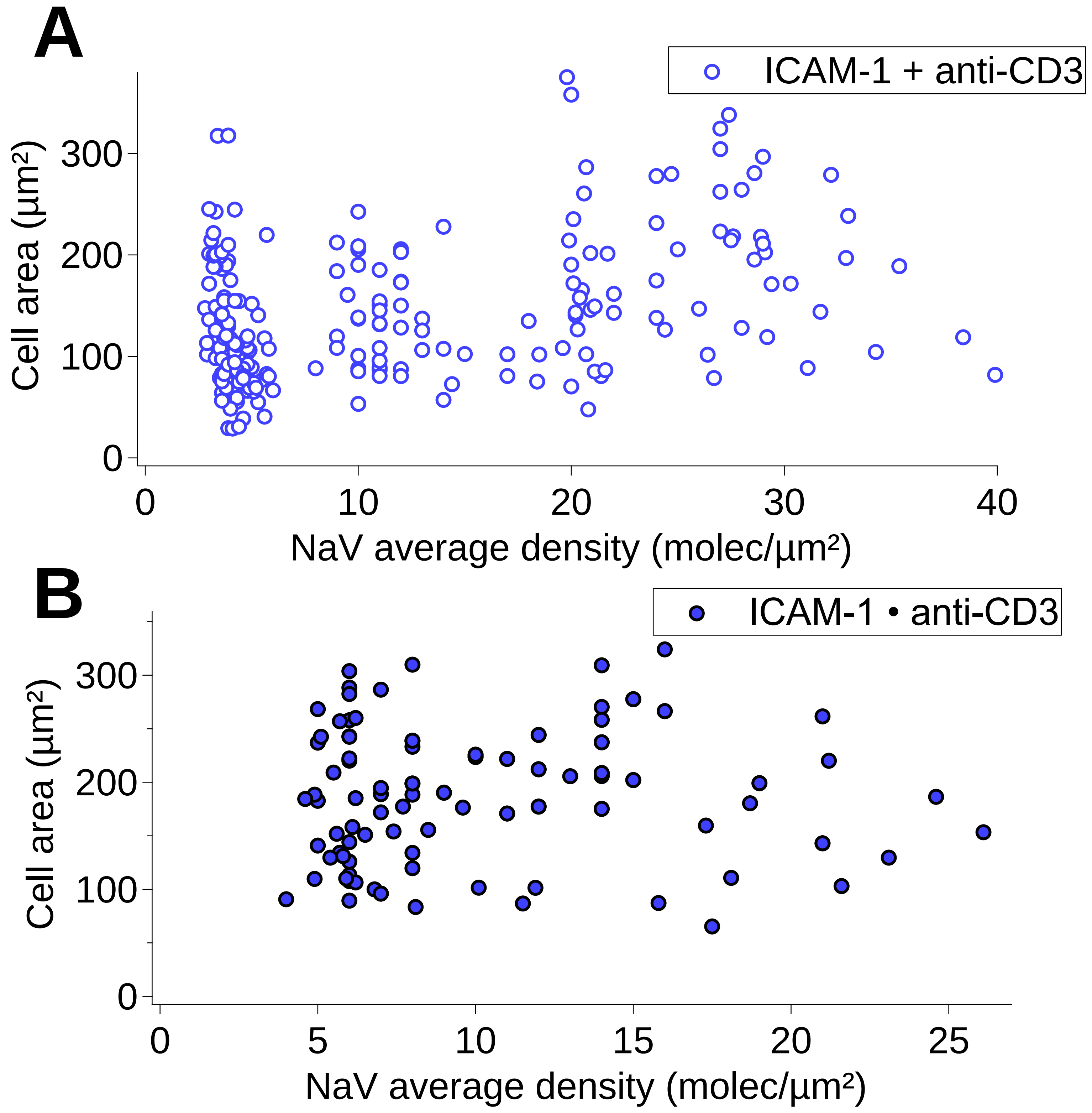

Fig S2B : **Impact of NaV density on cell spreading area.** Cell adhesion area as a function of the average NaV density over the whole sample, on homogeneous (**A**) or patterned (**B**) substrates fonctionnalized with anti-CD3 and ICAM-1. Each point represent one cells.

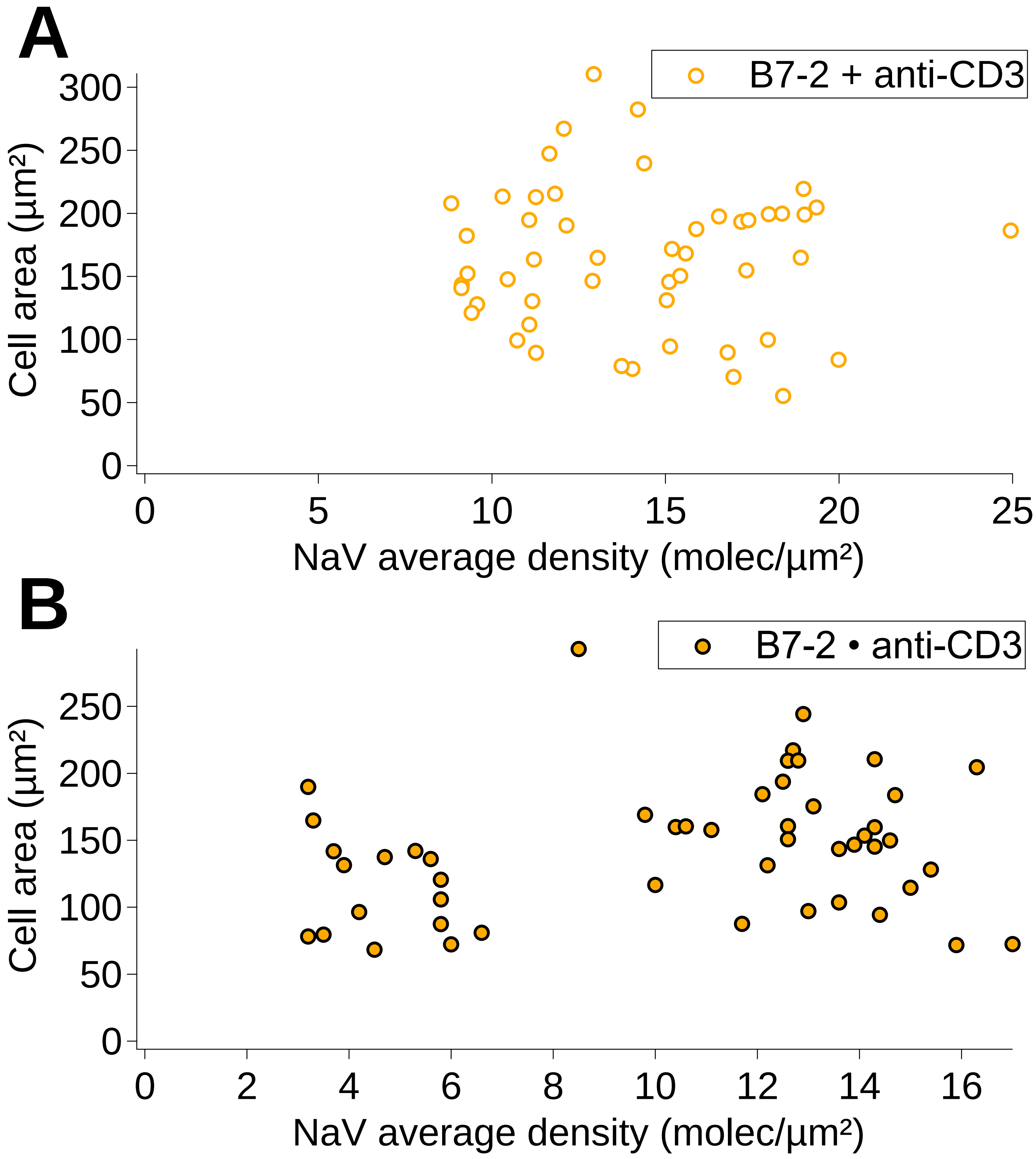

Fig S2C : **Impact of NaV density on cell spreading area.** Cell adhesion area as a function of the average NaV density over the whole sample, on homogeneous (**A**) or patterned (**B**) substrates fonctionnalized with anti-CD3 and B7-2. Each point represent one cells.

TABLE OF MEAN AND MEDIAN						
	N,n	Mean (µm²)	s.d (µm²)	SEM (µm²)	Median (µm²)	MAD (µm²)
**Bare+AntiCD3**	4, 75	148	57	7	130	26
**Bare•AntiCD3**	8, 109	207	95	9	170	62
**ICAM+AntiCD3**	7, 164	160	68	5	147	49
**ICAM•AntiCD3**	12, 271	298	153	9	259	78
**B7+AntiCD3**	2, 50	164	57	8	165	35
**B7•AntiCD3**	4, 203	160	62	4	156	45

Table S1. **An overview of the analysed data**. N and n signify number of samples and number of cells. SEM is standard error of mean and MAD is median absolute deviation.

HISTOGRAM COMPARING EACH HOMOGENEOUS SUBSTRATE WITH CORRESPONDING PATTERN

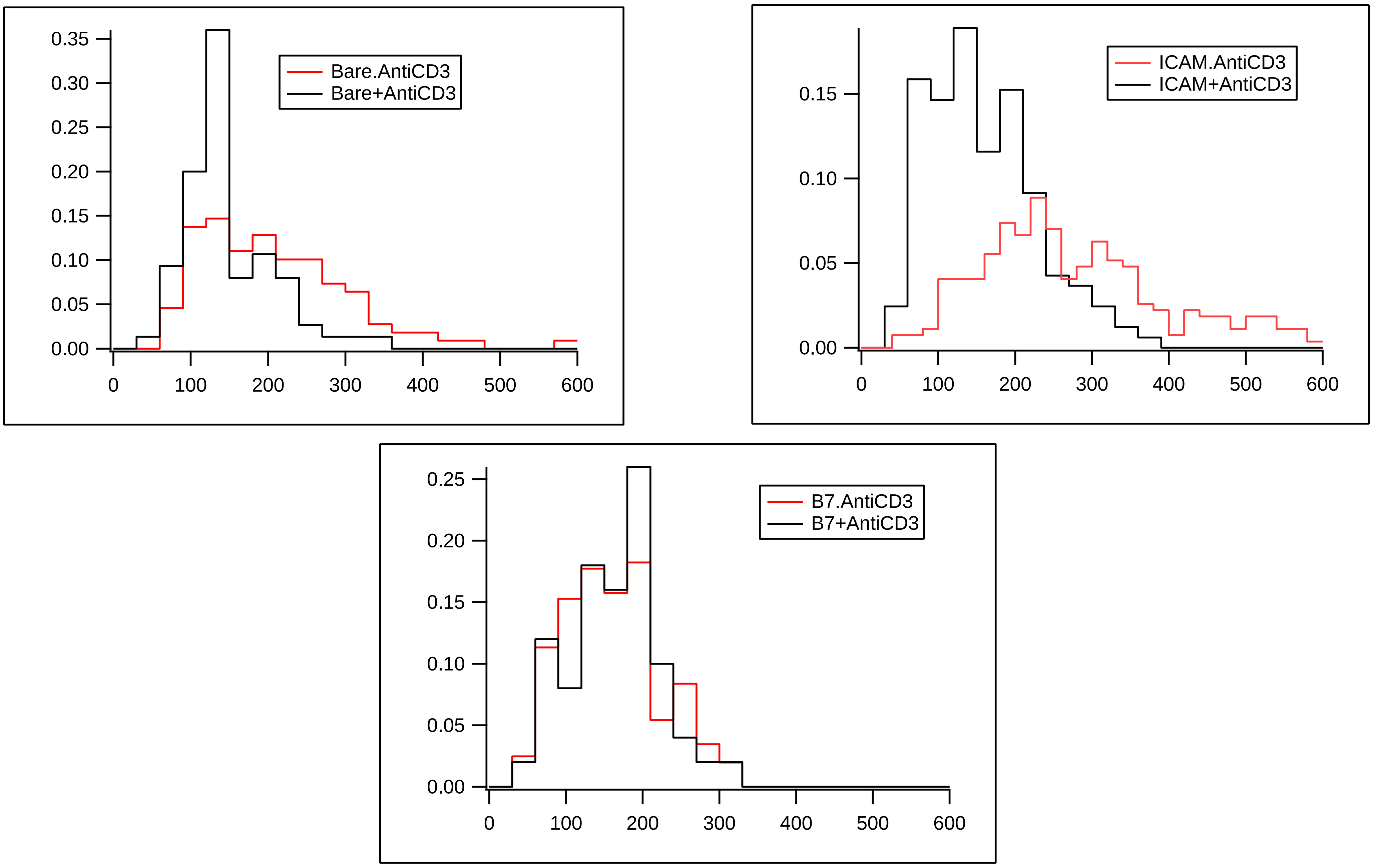

Fig S3 : **Histograms of the data presented in table S1, comparing patterned substrates with their homogeneous counterparts**.

p-values from Mann-whitney analysis						
	Bare+AntiCD3	Bare•AntiCD3	ICAM+AntiCD3	ICAM•AntiCD3	B7+AntiCD3	B7•AntiCD3
Bare+AntiCD3		**5.37e-06**	0.24	0	0.05	0.1
Bare•AntiCD3			3.60e-05	3.45e-09	0.01	3.89e-05
ICAM+AntiCD3				**0**	0.41	0.69
ICAM•AntiCD3					4.90e-13	0
B7+AntiCD3						**0.51**
B7•AntiCD3						

DIFFERENCES IN MEAN						
	Bare+AntiCD3	Bare•AntiCD3	ICAM+AntiCD3	ICAM•AntiCD3	B7+AntiCD3	B7•AntiCD3
Bare+AntiCD3		**60**	12	150	17	13
Bare•AntiCD3			47	91	43	47
ICAM+AntiCD3				**138**	4	0.2
ICAM•AntiCD3					134	138
B7+AntiCD3						**4**
B7•AntiCD3						
**DIFFERENCES IN MEDIAN**						
	Bare+AntiCD3	Bare•AntiCD3	ICAM+AntiCD3	ICAM•AntiCD3	B7+AntiCD3	B7•AntiCD3
Bare+AntiCD3		**60**	17	129	35	26
Bare•AntiCD3			42	69	25	34
ICAM+AntiCD3				**111**	17	8
ICAM•AntiCD3					94	103
B7+AntiCD3						**9**
B7•AntiCD3						

Table S2**. Pair-wise comparison of the substrates**. The reported p-values are from 2 tailed Wilcoxon-Mann-Whitney Rank Test (performed using IGOR-PRO software). $p<0.0001$ are coloured green (extremely likely to be different) and those >0.1 are coloured blue (extremely likely to be same). To quantify the size of the difference (effect-size) we report the differences in mean and median, the latter being more appropriate for the non-normal distributions reported here. In each case, the most relevant comparison is between the substrates with patterned-ligands and the corresponding homogeneous substrate, accordingly, these reported values are highlighted in bold. Note that the numbers are meaningful only when the distributions are deemed to be different (green cells).

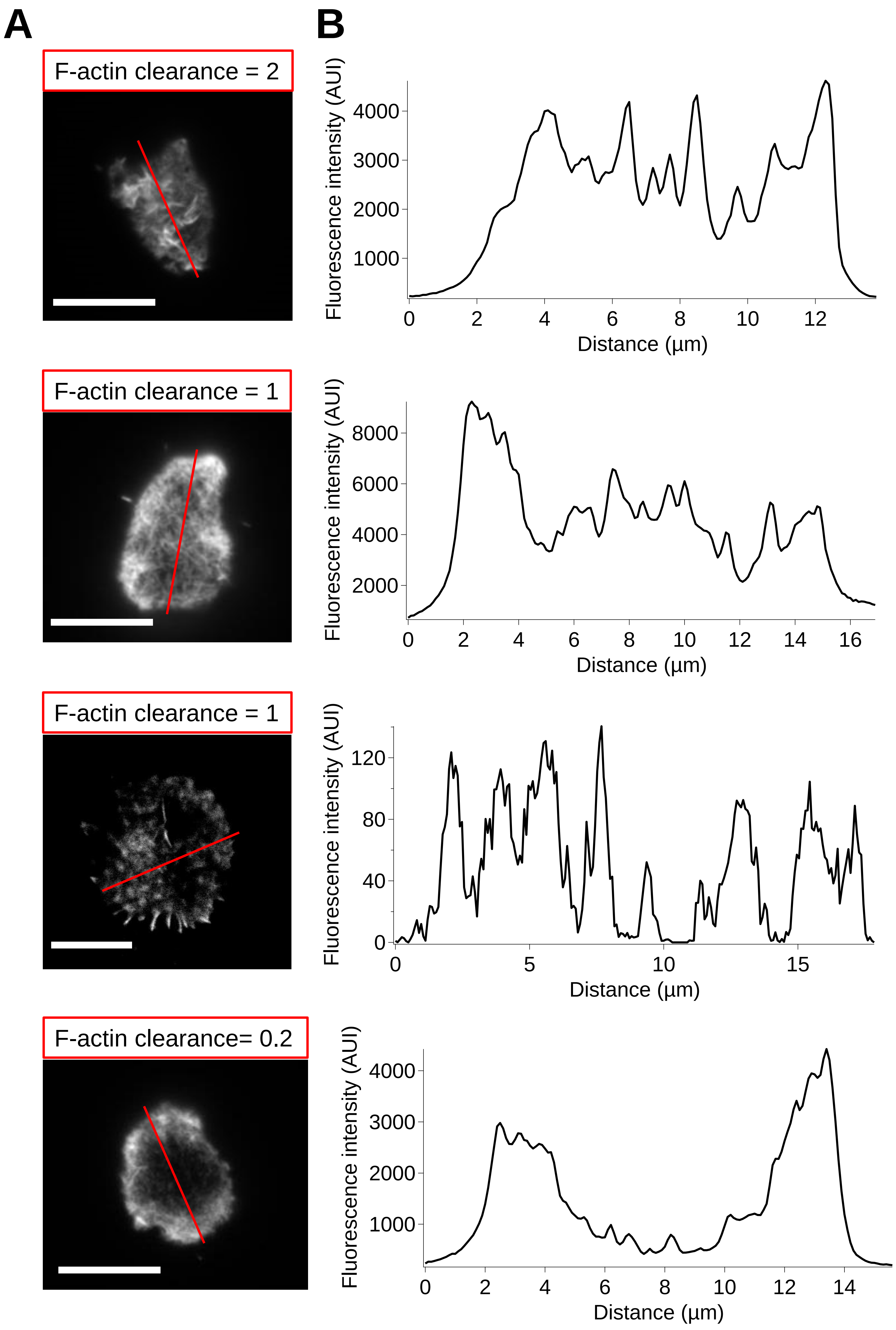

Fig S4: **Examples of the different F-actin architectures reported**. **A-** Exemplary fluorescence image with the corresponding calculated F-actin clearance parameter mentioned at the top. **B-** Corresponding intensity profile along the red line. Scale bare = 10µm.

A

TCR cluster area (µm²)	Number of clusters: Bare +anti-CD3	Bare •anti-CD3	p-value
[0;0.1]	1252	993	0
[0.1;0.2]	1068	1250	0
[0.2;0.3]	350	616	6.00e-4
[0.3;0.4]	154	443	0
[0.4;0.5]	75	298	0
**[0.5;0.6]**	**63**	**209**	**0**
[0.6;0.7]	36	147	0
[0.7;0.8]	20	107	0
[0.8;0.9]	16	89	0
[0.9;1.0]	19	58	0
[1.0;1.1]	16	55	1.30e-3
[1.1;1.2]	6	39	1.00e-4
[1.2;1.3]	6	23	2.41e-2
[1.3;1.4]	5	17	7.68e-2
[1.4;1.5]	7	18	1.77e-1
[1.5;1.6]	3	11	1.31e-1
[1.6;1.7]	0	11	5.40e-3
Total number of cluster	3104	4416	

TCR cluster area (µm²)	Number of clusters: ICAM-1 +anti-CD3	ICAM-1 •anti-CD3	p-value
[0;0.1]	234	452	1.70e-3
[0.1;0.2]	271	411	0
[0.2;0.3]	107	221	1.76e-1
[0.3;0.4]	56	125	6.47e-1
[0.4;0.5]	18	102	4.00e-4
**[0.5;0.6]**	**14**	**78**	2.30e-3
[0.6;0.7]	8	51	6.80e-3
[0.7;0.8]	5	56	2.00e-4
[0.8;0.9]	4	57	1.00e-4
[0.9;1.0]	2	48	1.00e-4
[1.0;1.1]	4	42	1.80e-3
[1.1;1.2]	3	28	1.51e-2
[1.2;1.3]	1	25	4.00e-3
[1.3;1.4]	5	16	5.70e-1
[1.4;1.5]	3	9	7.35e-1
[1.5;1.6]	1	11	1.08e-1
[1.6;1.7]	2	19	4.43e-2
Total number of clusters	744	1783	

TCR cluster area (µm²)	Number of clusters: B7-2 +anti-CD3	B7-2 •anti-CD3	p-value
[0;0.1]	1192	129	0
[0.1;0.2]	932	160	4.76e-1
[0.2;0.3]	291	58	1.42e-1
[0.3;0.4]	132	42	1.00e-4
[0.4;0.5]	79	17	2.94e-1
**[0.5;0.6]**	**67**	**10**	7.88e-1
[0.6;0.7]	36	14	4.20e-3
[0.7;0.8]	32	9	1.42e-1
[0.8;0.9]	26	4	9.11e-1
[0.9;1.0]	30	3	4.11e-1
[1.0;1.1]	5	2	2.68e-1
[1.1;1.2]	1	4	0
[1.2;1.3]	6	4	1.79e-2
[1.3;1.4]	1	1	1.43e-1
[1.4;1.5]	3	1	5.28e-1
[1.5;1.6]	2	0	5.68e-1
[1.6;1.7]	1	1	1.43e-1
Total number of clusters	2841	464	

different proportion

same proportion

B

ZAP-70 cluster area (µm²)	Number of clusters: Bare •anti-CD3	ICAM-1 •anti-CD3	p-value
[0;0.1]	53	50	1.32e-1
[0.1;0.2]	336	399	0
[0.2;0.3]	221	417	0
[0.3;0.4]	216	187	2.86e-2
[0.4;0.5]	214	99	3.00e-4
**[0.5;0.6]**	**231**	**83**	**0**
[0.6;0.7]	185	62	0
[0.7;0.8]	152	29	0
[0.8;0.9]	137	46	0
[0.9;1.0]	80	19	0
[1.0;1.1]	76	25	6.00e-4
[1.1;1.2]	51	19	1.49e-2
[1.2;1.3]	56	11	0
[1.3;1.4]	34	15	1.25e-1
[1.4;1.5]	28	12	1.43e-1
[1.5;1.6]	20	8	1.69e-1
[1.6;1.7]	10	6	7.55e-1
Total number of clusters	2214	1570	

ZAP-70 cluster area (µm²)	Number of clusters: Bare •anti-CD3	B7-2 •anti-CD3	p-value
[0;0.1]	53	294	0
[0.1;0.2]	336	827	0
[0.2;0.3]	221	601	0
[0.3;0.4]	216	305	1.68e-1
[0.4;0.5]	214	197	9.00e-4
**[0.5;0.6]**	**231**	**124**	**0**
[0.6;0.7]	185	84	0
[0.7;0.8]	152	63	0
[0.8;0.9]	137	61	0
[0.9;1.0]	80	48	0
[1.0;1.1]	76	18	0
[1.1;1.2]	51	18	0
[1.2;1.3]	56	12	0
[1.3;1.4]	34	8	0
[1.4;1.5]	28	9	1.00e-4
[1.5;1.6]	20	5	3.00e-4
[1.6;1.7]	10	3	1.77e-2
Total number of clusters	2214	2721	

ZAP-70 cluster area (µm²)	Number of clusters: ICAM-1 •anti-CD3	B7-2 •anti-CD3	p-value
[0;0.1]	50	294	0
[0.1;0.2]	399	827	3.50e-3
[0.2;0.3]	417	601	1.00e-4
[0.3;0.4]	187	305	3.02e-1
[0.4;0.5]	99	197	3.60e-1
**[0.5;0.6]**	**83**	**124**	1.98e-1
[0.6;0.7]	62	84	9.28e-2
[0.7;0.8]	29	63	3.75e-1
[0.8;0.9]	46	61	1.22e-1
[0.9;1.0]	19	48	1.94e-1
[1.0;1.1]	25	18	2.30e-3
[1.1;1.2]	19	18	4.92e-2
[1.2;1.3]	11	12	2.32e-1
[1.3;1.4]	15	8	3.30e-3
[1.4;1.5]	12	9	4.20e-2
[1.5;1.6]	8	5	5.37e-2
[1.6;1.7]	6	3	5.42e-2
Total number of clusters	1570	2721	

different proportion

same proportion

SI Table 3: **Quantification of cluster-size comparison between patterned and homogeneous substrates by proportion's z-test** (performed using in-build function in Microsoft Office Excel software). **A-** TCR clusters and **B-** ZAP-70 clusters. Both in presence and absence of ICAM-1, the patterned substrates are enriched in TCR-clusters of size ranging from about 0.4 to 1.3 µm². For B7.2, enrichment is less clear. ZAP-70-clusters are also enriched both in presence and absence of ICAM-1, and to a lesser extent in presence of B7.2. Note that ZAP-70 clusters of sizes > 1.3 µm² are enriched in presence, but not in absence of ICAM-1, implying that presence of ICAM-1 may support larger ZAP-70 clusters. When the difference is significant, the cell is coloured green, dark when clusters are enriched on patterns and light green otherwise.

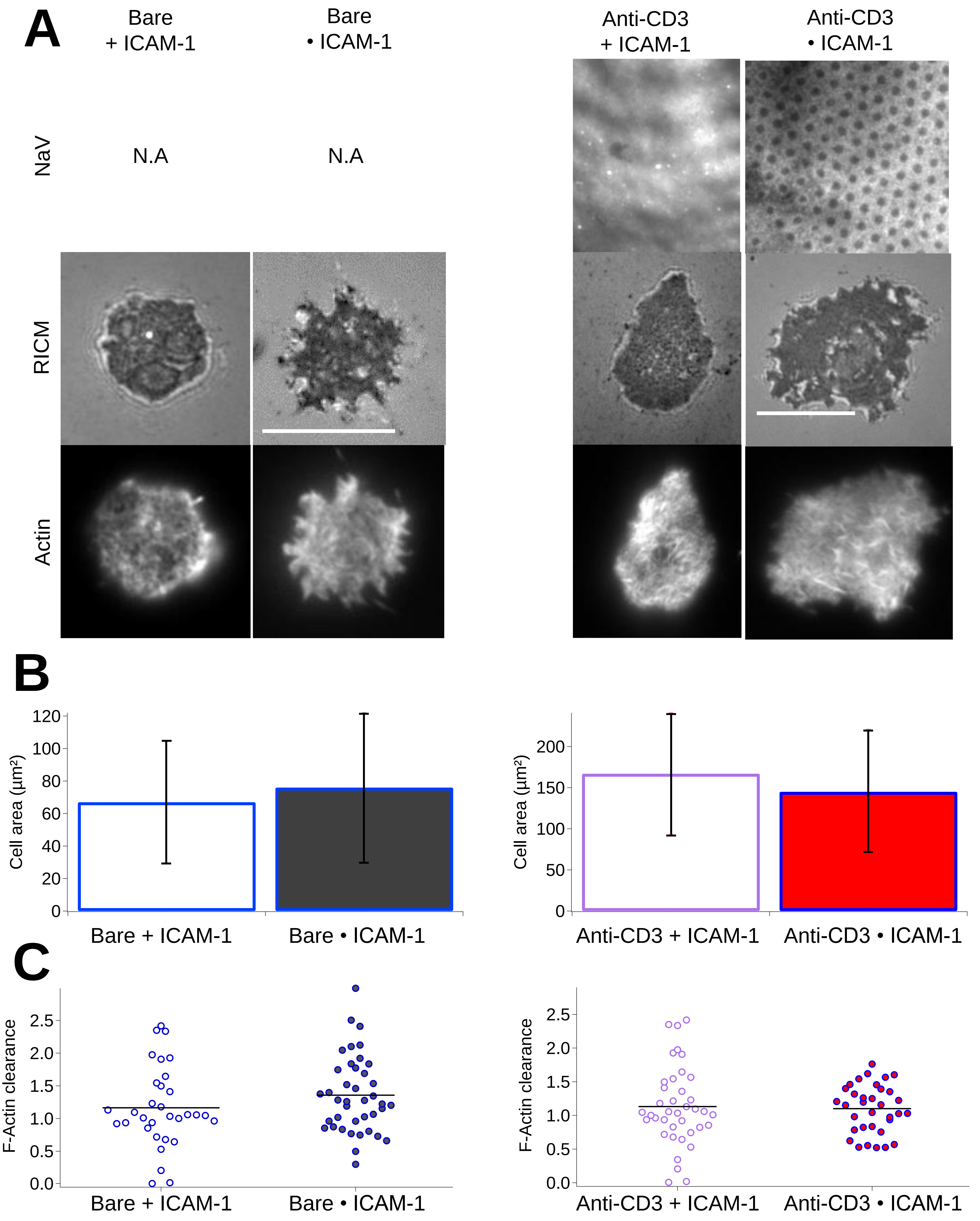

Fig S5: **Impact of ICAM-1 clustering in presence or not of anti-CD3**. Cells were allowed tointeract with homogeneous and patterned substrate in presence of only ICAM-1 (left panel) or of ICAM-1 and anti-CD3 (right panel). The cells were then fixed and labeled with a fluorescent Phalloidin. **A-** T-cell adhesion and actin organization. Top row: epi-fluorescent images of the underlaying organisation of NaV. middle row: RICM images of a Jurkat T-cell after 30 minutes engagement on the substrate. Bottom row: TIRFM images of the marked cells. Scale bare = 10µm. **B-** Spreading area of Jurkat T-cells on the substrates. Error bars represent standard deviations. **C-** Scatter dot plot of the F-actin clearance.

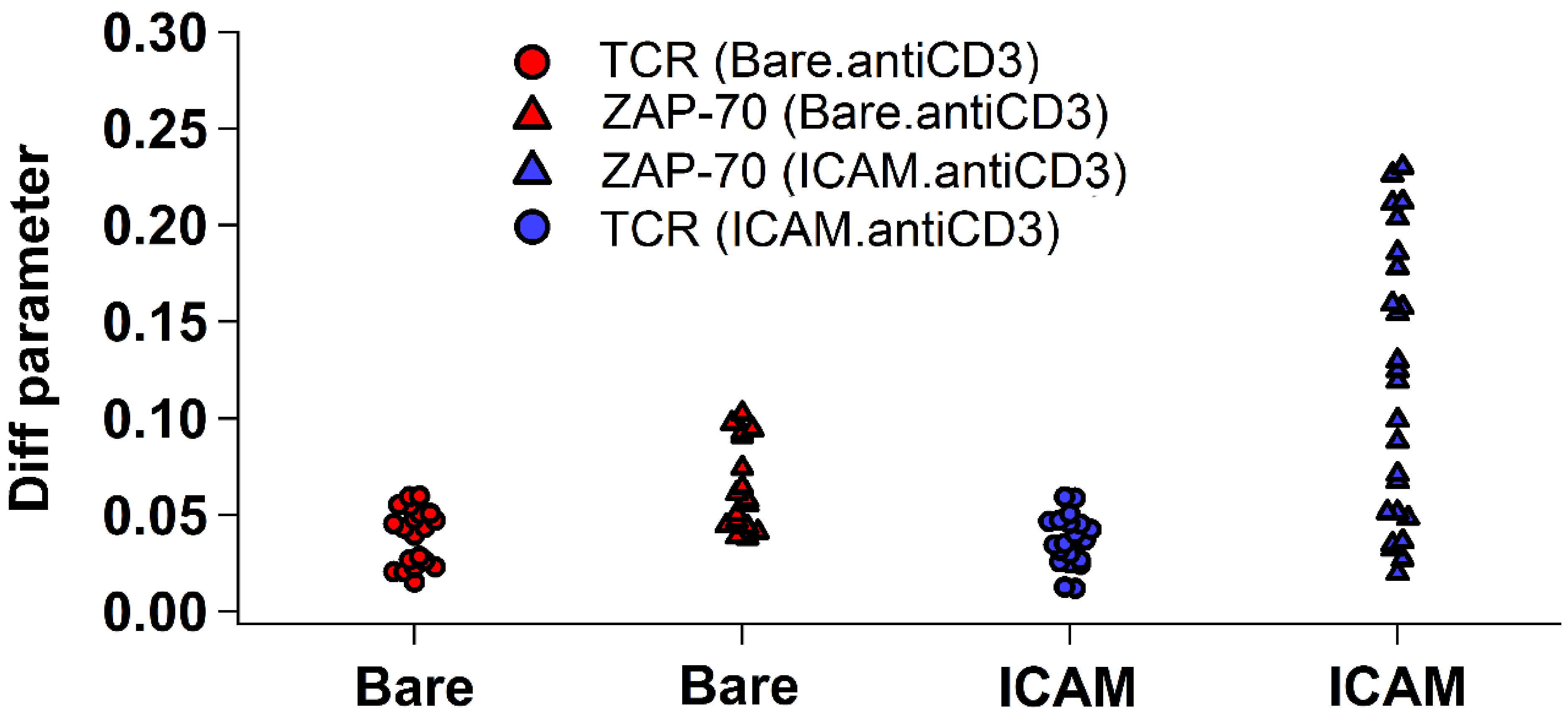

Fig S6: **Comparison between TCR and ZAP-70 clusters in terms of their overlap with the pattern**. Intensity profiles of the ligand-dots (from epi-images of NAV) were plotted along with the corresponding intensity profile of the protein clusters that may be present on top (from TIRF images of TCR/ZAP-70). Each profile, corresponding to a single dot/cluster (delimitation was based on the NAV pattern, using the mid-point between two adjacent dots as extremity), was normalized to obtain the profiles $I(x)^{NAV}$ and $I(x)^{TCR/ZAP}$. Each pair $I(x)^{NAV}$ and $I(x)^{TCR}$ or $I(x)^{NAV}$ and $I(x)^{ZAP}$, a difference-squared profile $D(x) = [I(x)^{NAV} - I(x)^{TCR/ZAP}]^2$ was calculated. The diff parameter quantifies the rms (root mean square) difference from D(x) by summing over all the points, taking the square root and dividing by the length of the profile to account for size differences. More the overlap between $I(x)^{NAV}$ and $I(x)^{TCR/ZAP}$, lower is Diff parameter; conversely, a large difference in the NAV-dot and TCR/ZAP cluster profile gives a large Diff parameter. It is seen that both in presence and absence of ICAM-1, the ZAP-70 clusters overlap less than the TCR clusters and that this effect is more pronounced in the presence of ICAM-1.

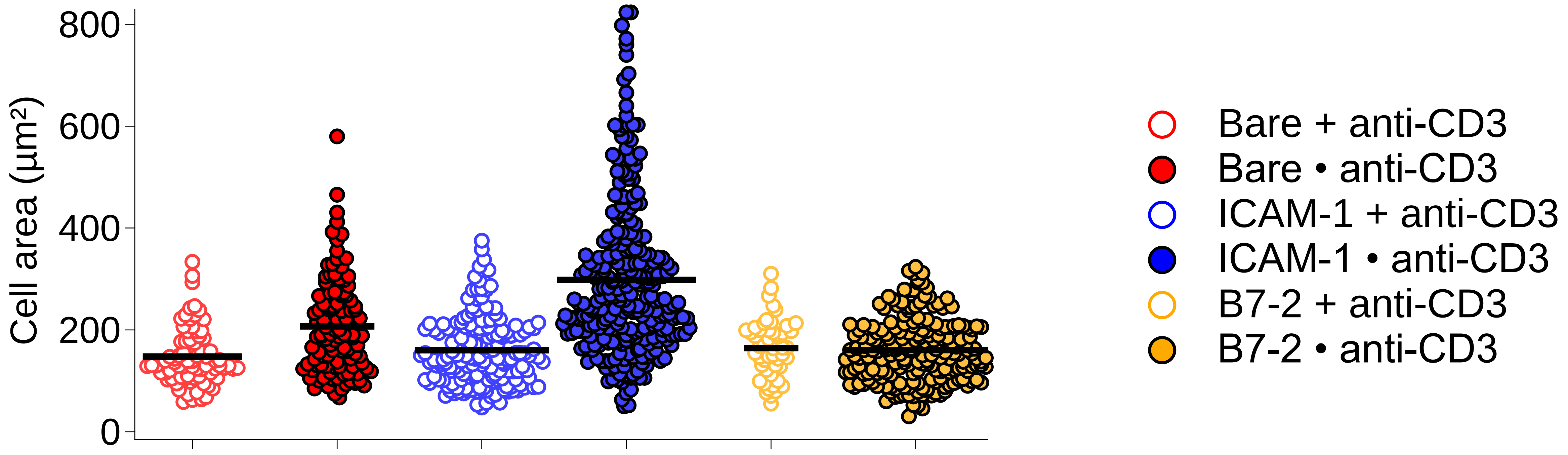

Fig S7 A : **Scatter-dot plot of cell spreading area measured from RICM images for all the substrates described in Table S1 and S2**. (data gathered from main-text Figs).

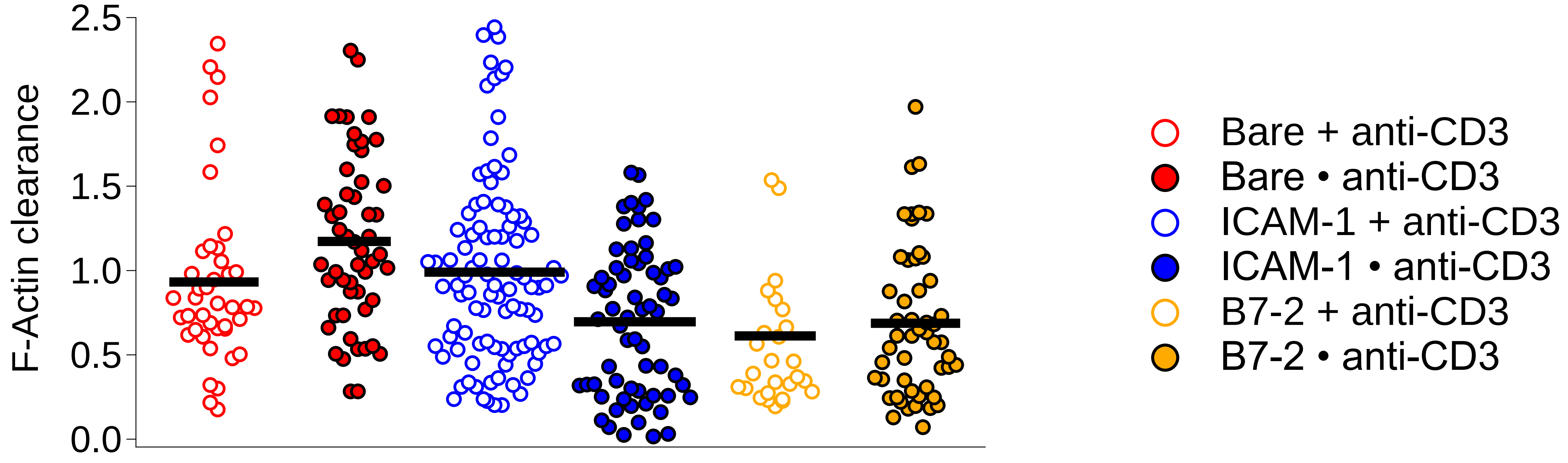

Fig S7B : **Scatter-dot plot of the F-actin clearance calculated from TIRFM images for all the substrates** (data gathered from main-text Figs. 2B&C, 3B&C, and 4B&C). Black-bars are averages.

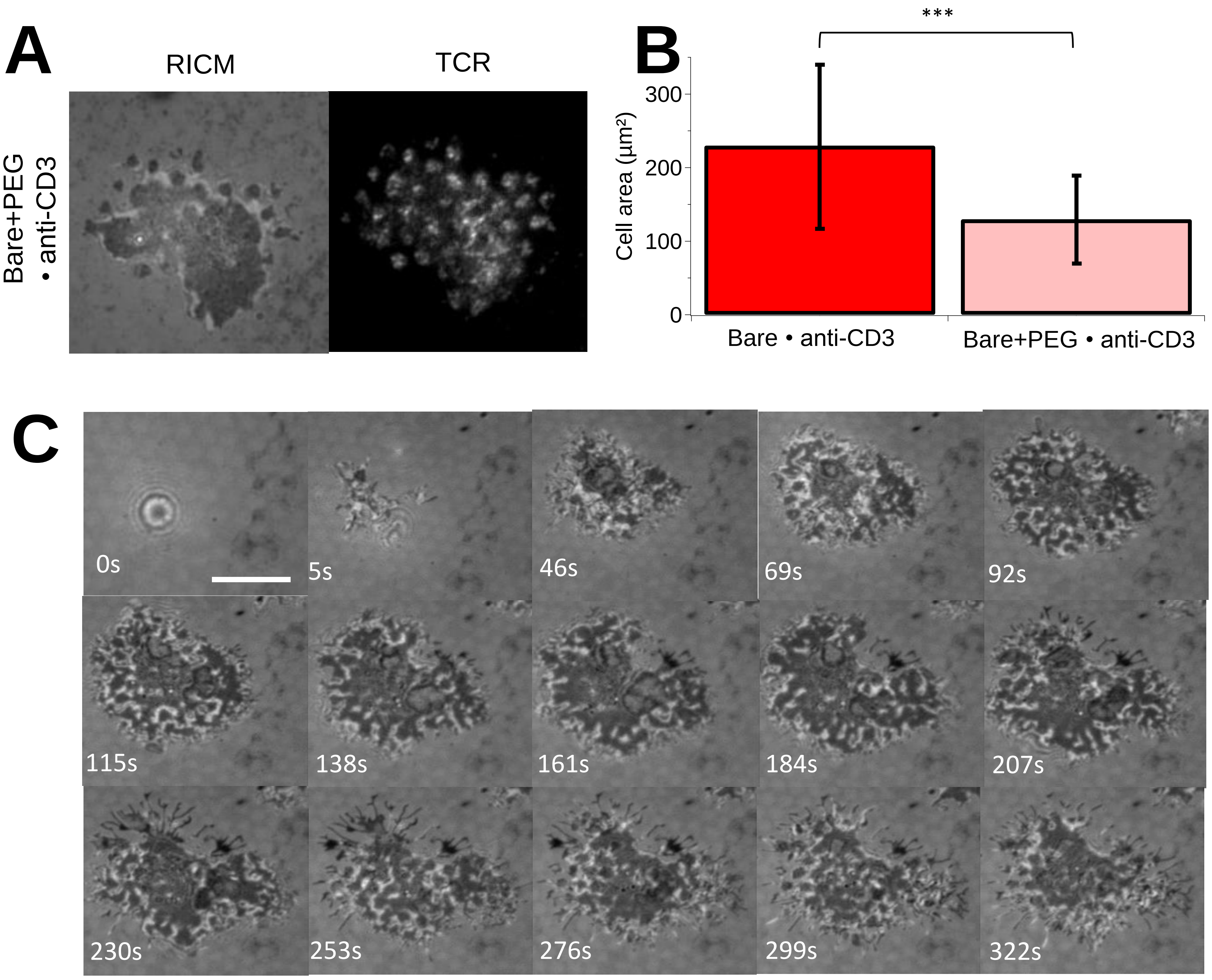

Fig S8 : **Impact of anti-CD3 clustering when SLB is additionally doped with PEG-carrying lipids**. **A-** RICM images and TCR clusters of T-cells spreading on patterned substrates with nano-dots of anti-CD3 surrounded by non-functionalized SLB and/or PEG. **B-** Spreading area of Jurkat T-cells on substrates with or without PEG in the SLB. Error bars represent standard deviations. ***=p<0.001. **C-** Dynamic of cell spreading area of Jurkat T-cell measured on RICM image sequence during cells engagement on Bare+PEG anti-CD3.

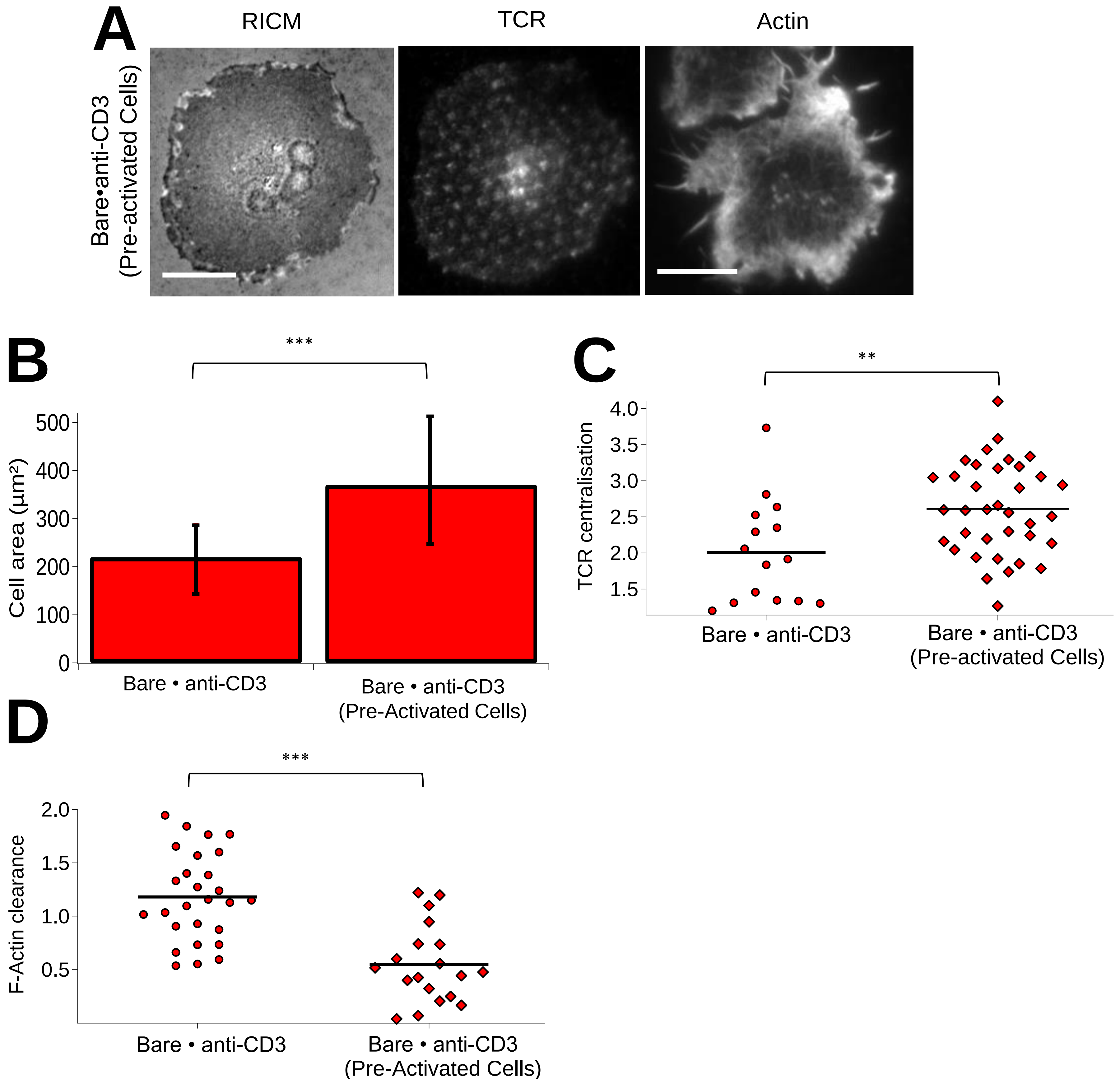

Fig S9 : **Impact of anti-CD3 clustering on T-cells with a pre-labelling of the TCR with an anti-body (anti-V8). A-** RICM images and TIRFM images of TCR clusters and actin of T-cells spreading on patterned substrates with nano-dots of anti-CD3 surrounded by non-functionalized SLB .Scale Bar =10µm. **B-** Spreading area of Jurkat T-cells on Bare • anti-CD3 substrates with or without TCR pre-labelling. Error bars represent standard deviations. **C-** Scatter dot plot of the TCR centralisation. **D-** Scatter dot plot of the F-actin clearance. . ***=p<0.001 and **=0.001<p<0.01.
